# Personalizing motion sickness models: estimation and statistical modeling of individual-specific parameters

**DOI:** 10.3389/fnsys.2025.1531795

**Published:** 2025-06-16

**Authors:** Varun Kotian, Daan M. Pool, Riender Happee

**Affiliations:** ^1^Faculty of Mechanical Engineering, Cognitive Robotics, Delft University of Technology, Delft, Netherlands; ^2^Faculty of Aerospace Engineering, Control and Simulation, Delft University of Technology, Delft, Netherlands

**Keywords:** motion sickness, simulator sickness, modeling, driving simulators, automated vehicles

## Abstract

As users transition from drivers to passengers in automated vehicles, they often take their eyes off the road to engage in non-driving activities. In driving simulators, visual motion is presented with scaled or without physical motion, leading to a mismatch between expected and perceived motion. Both conditions elicit motion sickness, calling for enhanced vehicle and simulator motion control strategies. Given the large differences in sickness susceptibility between individuals, effective countermeasures must address this at a personal level. This paper combines a group-averaged sensory conflict model with an individualized Accumulation Model (AM) to capture individual differences in motion sickness susceptibility across various conditions. The feasibility of this framework is verified using three datasets involving sickening conditions: (1) vehicle experiments with and without outside vision, (2) corresponding vehicle and driving simulator experiments, and (3) vehicle experiments with various non-driving-related tasks. All datasets involve passive motion, mirroring experience in automated vehicles. The preferred model (AM2) can fit individual motion sickness responses across conditions using only two individualized parameters (gain *K*_1_ and time constant *T*_1_) instead of the original five, ensuring unique parameters for each participant and generalisability across conditions. An average improvement factor of 1.7 in fitting individual motion sickness responses is achieved with the AM2 model compared to the group-averaged AM0 model. This framework demonstrates robustness by accurately modeling distinct motion and vision conditions. A Gaussian mixture model of the parameter distribution across a population is developed, which predicts motion sickness in an unseen dataset with an average RMSE of 0.47. This model reduces the need for large-scale population experiments, accelerating research and development.

## 1 Introduction

Automated vehicles and driving simulators are very different technologies. However, they both share two common facts. The first is that they have become very popular in recent years, a trend that is expected to continue in the future. Secondly, they both share a common issue in *motion sickness*. Users of automated vehicles will move away from being drivers to passengers, preferably engaged in other activities such as reading or using laptops and smartphones. In driving simulators, realistic (unscaled) visual motion is presented with scaled or even without any physical motion. This causes a mismatch between expected and perceived motion, eliciting motion sickness (Bos et al., 2020). Even though these two examples are different and referred to as car and simulator sickness, respectively, the inherent mechanism that causes motion sickness in both, i.e., *sensory-expectancy conflict*, is the same (Reason, 1978).

The mechanisms behind the development and evolution of motion sickness have been studied extensively, relying heavily on models that predict sensory conflicts based on mathematical models of the vestibular and visual sensory systems (Bos and Bles, 1998; Wada et al., 2020; Liu et al., 2022; Irmak et al., 2023; Kotian et al., 2024). In this paper, these models are referred to as “conflict generation” models. However, these models generally predict group-averaged sickness development in terms of the Motion Sickness Incidence (MSI), which does not directly reflect some crucial dynamics of motion sickness development, such as recovery and hypersensitivity (Oman, 1990; Irmak et al., 2023). Furthermore, such group-averaged models cannot be reliably used for predicting motion sickness at an individual level.

**Figure 1:**
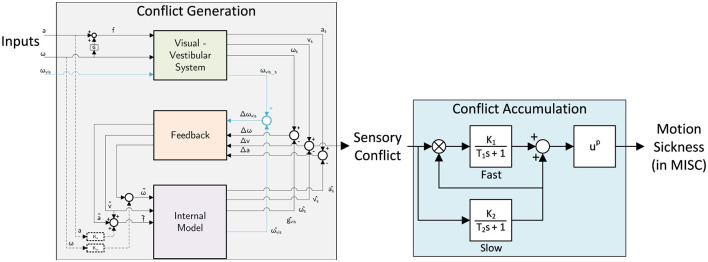
Framework for combined “conflict generation” using the SVC model (Wada et al., 2020; Kotian et al., 2024) and “conflict accumulation” (Oman, 1990; Irmak et al., 2022) model to predict individual motion sickness, i.e., MIsery SCale (Bos et al., 2005).

In this paper, we aim to combine an available group-average “conflict generation” model—the Subjective Vertical Conflict (SVC) model by Wada et al. (2020)— with an individualized “conflict accumulation” model (AM) as also used by Irmak et al. (2020) to improve the ability to capture the differences in individual motion sickness susceptibility. This also requires the use of an individual motion sickness metric, such as the MIsery Scale (Bos et al., 2005), instead of a group-averaged metric like Motion Sickness Incidence (MSI). The proposed modeling framework is shown in Figure 1. The conflict accumulation part is a modified form of the model by Oman (1990). The accumulation model is nonlinear and has conflict in *m*/*s*^2^ as input, and the output is unitless in MISC (defined in Section 6 of Supplementary material). This complicates the definition of units for the gains due to the multiplication and the power term. For conflicts generated by motion accelerations in one degree-of-freedom, the personalisation of sickness accumulation parameters has already been shown to improve modeling accuracy by a factor of 2 compared to using group-averaged parameters (Irmak et al., 2020). Our work extends this approach to full 6 degrees-of-freedom motion perceived from visual and vestibular inputs, which requires a “conflict generation” model to account for realistic sensory conflict predictions, as in Figure 1. Further details regarding this model structure will be provided in Section 2.

The goal of the paper is to demonstrate the feasibility of this combined motion sickness model approach—i.e., combining a group-average “conflict generation” and a personalized “conflict accumulation” model—for capturing individual differences in motion sickness susceptibility. While evidence exists of individual differences in “conflict generation” model parameters as well (Irmak et al., 2021), quantifying these would require individual perception experimental data, which was not readily available (an exception is Irmak et al., 2021). Furthermore, we aim to find a “minimum effective” implementation of such a personalized motion sickness model by directly comparing different parameterisations of the “accumulation model.” For this, extending our own preliminary work in Kotian et al. (2023), this paper makes use of four existing datasets, see Table 1, where MISC was measured under sickening motion stimuli in (1) an experimental vehicle with and without out-of-the-window vision (Irmak et al., 2020), (2) vehicle experiments and matched driving simulator experiments (Talsma et al., 2023), (3) vehicle experiments with various non-driving related tasks (NDRTs) (Metzulat et al., 2024), and (4) on road vehicle experiments and sickness recreation experiments on a smaller track (Harmankaya et al., 2024). All datasets involve passive motion, representative of being driven by an automated vehicle, and test two or three different conditions with the same participants. Inoue et al. (2024) also report on a similar approach as used by Kotian et al. (2023), where only the pre- and post-scaling of the “conflict accumulation” model was varied between different participants. The resulting individualized models were validated using a 1 degree-of-freedom motion stimulus experiment with variation in head movement (Inoue et al., 2024). Our current work performs extended validation with full 6 degrees-of-freedom motion and with variations in visual and vestibular inputs in real driving scenarios, which requires a “conflict generation” model to account for realistic sensory conflict predictions. Furthermore, we perform an explicit optimization of the required number of parameters for personalizing the “conflict accumulation” model, including the model's gains and time constants instead of a simple pre- and post-scaling.

**Table 1:** Experimental datasets used in this study.

**Datasets**	**Details**	**Reference**	**No. of participants**
Slalom drive	Slalom with internal and external vision Motion sickness responses for hypersensitivity	Irmak et al., 2020	16
Car and simulator	Naturalistic drive in vehicle and moving base simulator Only external vision	Talsma et al., 2023	24
NDRT Drive	Naturalistic driving data with varying NDRTs Internal vision	Metzulat et al., 2024	20
Sickness Recreation	Naturalistic driving data on road recreated on a smaller track Internal vision	Harmankaya et al., 2024	47

This paper presents a direct comparison of fitting the proposed combined motion sickness model for all individual participants in the first three datasets listed in Table 1. For every participant, parameters are always estimated *across* the different conditions tested in each dataset to ensure a “minimum effective” and generalisable result. Additionally, using the individual-specific variations in the parameter values estimated from these datasets, a probabilistic Gaussian mixture model is used to capture the observed statistical variations. The capacity of this statistical modeling approach for predicting individual variations in motion sickness across a population is verified by predicting the final Sickness Recreation dataset, see Table 1.

By achieving these goals, this paper will show that our proposed modeling framework can be used for personalized motion sickness modeling for automated vehicles. The obtained model predictions can be used to optimize the comfort levels of individual automated vehicle users by adapting its driving style, i.e., by limiting the acceleration and rotations of the car they may be especially sensitive to. Furthermore, the derived statistical model can directly improve and accelerate the design and testing of new driving simulator motion cueing algorithms that aim to optimize simulator sickness, as in Hogerbrug et al. (2020), Baumann et al. (2021), and Jain et al. (2023).

## 2 Methods

### 2.1 Experimental datasets

For the analysis in this paper, we make use of four different published datasets, see Table 1. All datasets include measured sickness responses to passive road vehicle motion, representative of being driven by an automated vehicle. In these datasets, individual motion sickness levels were reported using the MISC scale as a function of time, and all showed major individual differences in sickness susceptibility.

**Figure 2:**
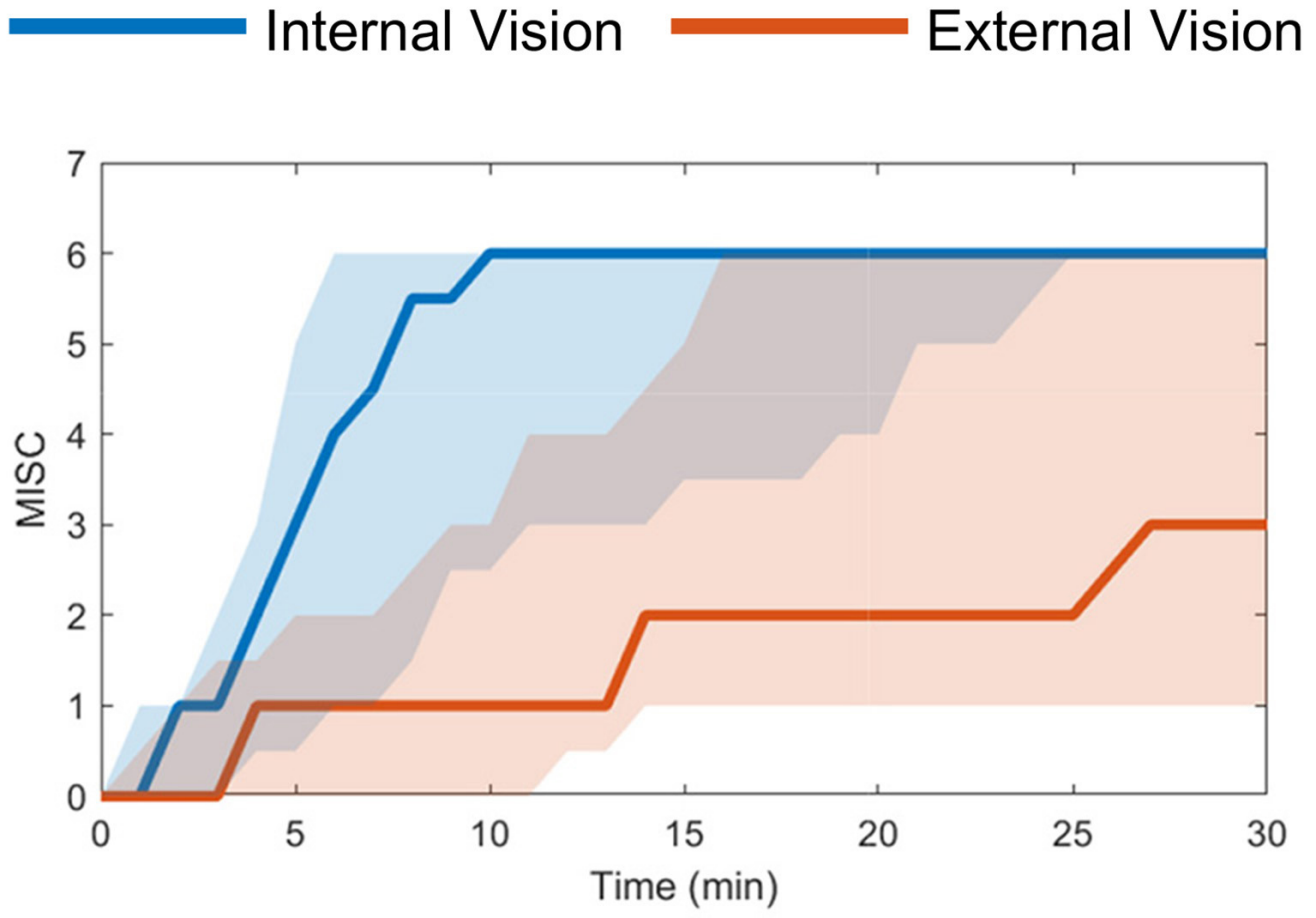
Slalom Drive dataset (Irmak et al., 2020) median MISC levels vs. time in the conditions of external (red) and internal vision (blue) with the shaded region showing the 25th to 75th percentiles.

First, the real-world “Slalom Drive” dataset includes varying vision conditions, i.e., with and without an outside view, where mean MISC levels at the end of motion exposure were 5.3 (severe symptoms) with an outside view and 3.3 (some symptoms) without an outside view (Experiment 1 in Irmak et al., 2020), see Figure 2. The experiment was terminated when a MISC value of 6 was reached. This experiment compared the motion sickness development with and without an outside view from the car. This dataset is ideal for proving that our new model framework can predict individual motion sickness for various vision conditions in real vehicles.

**Figure 3:**
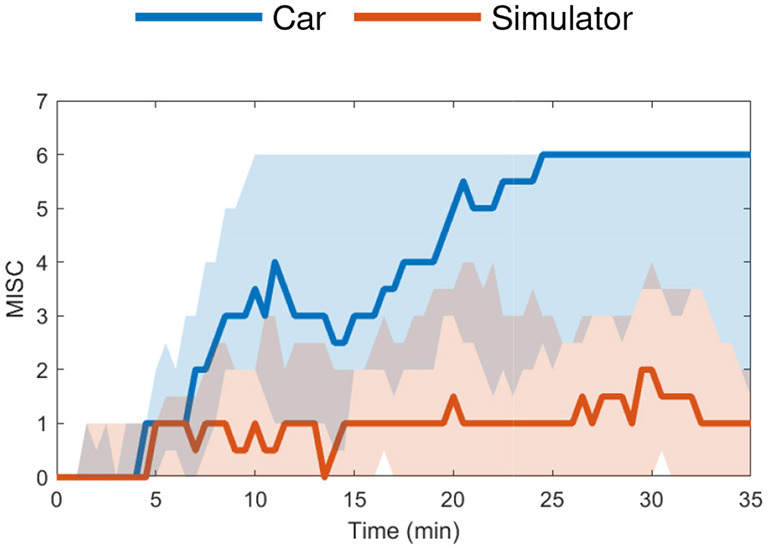
Car and Simulator dataset (Talsma et al., 2023) median MISC levels vs. time in the car (blue) and simulator (red) with the shaded region showing the 25th to 75th percentiles.

The “Car and Simulator” dataset (24 participants) contains motion sickness responses from real-world driving and its (matched) simulation on a moving-base driving simulator (Talsma et al., 2023). For this experiment, the mean MISC levels at the end of motion exposure were around 5.5 (severe symptoms) in the car and 1.5 (slight discomfort or vague symptoms) in the simulator, see Figure 3. The experiment was terminated when a MISC value of 6 was reached.

**Figure 4:**
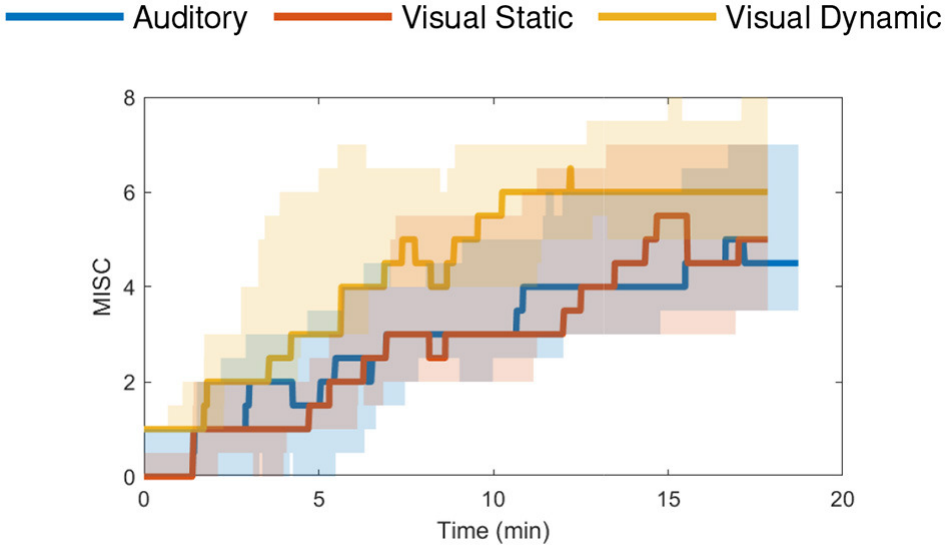
NDRT Drive dataset (Metzulat et al., 2024) median MISC levels vs. time in the three different tasks—auditory (blue), visual static (red) and visual dynamic (yellow) with the shaded region showing the 25th to 75th percentiles.

The “NDRT Drive” experiment was performed by 20 participants and focused on a real-world sickening drive around a fixed track with three different non-driving related tasks (NDRTs): visual dynamic, visual static and auditory. The mean MISC levels at the end of each condition were around 4.5 (medium symptoms) for the auditory, 5 (severe symptoms) for the visual static, and 6.5 (some nausea) for the visual dynamic condition, see Figure 4. This experiment was terminated when a MISC value of 7 was reached. However, several participants still showed a rapid increase to a MISC of 8 before or just after the experiment was actually stopped.

The “Sickness Recreation” experiment tested a method to efficiently replicate the on-road motion sickness of 47 participants on a smaller track. The mean MISC levels at the end of both drives were around 2 (vague symptoms) (Section 3.2).

In this paper, the “Slalom Drive,” “Car and Simulator,” and “NDRT Drive” datasets are used to demonstrate the capability of the individual modeling framework to generalize across different visual/vestibular input cases, whether these originate from a car or a simulator. Furthermore, the first three datasets are used to study the number of parameters needed to accurately model motion sickness development in different participants, while the “Sickness Recreation” dataset is used for validation of the statistical model over a new population.

### 2.2 Model framework: inputs and outputs

The structure of the model framework has been introduced in Section 1. As shown in Figure 1, inertial vehicle or simulator motion inputs—such as acceleration and angular velocity—as well as vision inputs—such as visual verticality (orientation) and visual rotation (rotational velocity)—are defined as the inputs of the “conflict generation” model, consistent with the SVC model by Wada et al. (2020) and Liu et al. (2022). For our analysis, we applied the full 6 degrees-of-freedom motion (3 translations and 3 rotations) as model inputs, using the recorded seat motion for the drives in the car for the “Car and Simulator,” “NDRT Drive” and “Sickness Recreation” dataset and recorded platform motion for the simulator in the “Car and Simulator” dataset, and recorded head motion for the “Slalom Drive” dataset. Example input data for all datasets is shown in Section 1 of Supplementary material.

The human eye estimates motion through vision by measuring the rotation of visual cues between the current and previous states, a process known as optic flow. We assume the visually perceived rotations to be equivalent to head (or vehicle, as in the case of the Car and Simulator dataset) rotations when observing the external environment. Consistent with Kotian et al. (2024), for internal vision, we select a zero visual input, assuming no visual head motion relative to the vehicle.

The output of the model is chosen to be the score on the MIsery SCale (MISC) by Bos et al. (2005), which quantifies the progression of sickness-related symptoms and has a positive relation to discomfort (De Winkel et al., 2022; Reuten et al., 2020). The MISC is an 11-point symptom-based scale that measures in discrete symptoms running from 0 to 10, where 0 means no symptoms and 10 stands for emesis (vomiting). In contrast to the discrete MISC scale, the sickness model predicts on a continuous scale. For model fitting, these continuous model predictions are compared with experimentally reported discrete MISC scores.

### 2.3 Model framework: model structures

As previously discussed, we combine two models in our proposed model framework. The first component of the model is the “conflict generation” model (see Figure 1, left), which models the integration of 6 degrees-of-freedom sensory inputs and generates a 1 degrees-of-freedom (scalar) sensory conflict signal. For our application, this model shall be reliable in forecasting conflict signals, especially for 6 degrees-of-freedom (automated) road vehicle motion. Additionally, the model must include explicit visual inputs so that the effects of different vision conditions can be predicted. To select an appropriate model, we refer to our previous study (Kotian et al., 2024), where different “conflict generation” models and implementations of vision inputs were directly compared.

**Figure 5:**
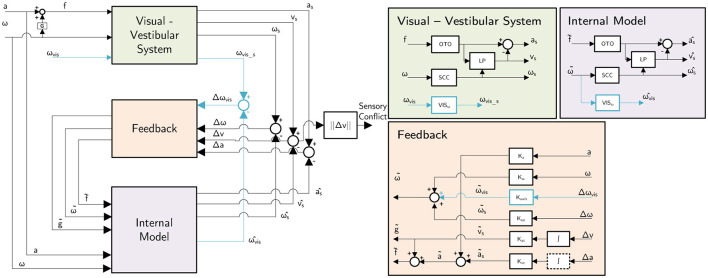
The selected “conflict generation” model, which is the SVC model as in Kotian et al. (2024) based on the model by Wada et al. (2020).

Based on Kotian et al. (2024), we select the Subjective Vertical Conflict (SVC) model with only a visual rotational velocity input as proposed in Wada et al. (2020) for predicting a subjective vertical conflict (a 3-dimensional vector) that drives motion sickness, see Figure 5. This model has been shown to accurately replicate the frequency and amplitude dynamics of motion sickness as reported in numerous previous studies (McCauley et al., 1976; Golding and Markey, 1996; Griffin and Mills, 2002; Howarth and Griffin, 2003; Irmak et al., 2021, 2022). Kotian et al. (2024) show that the visual vertical input that has also been proposed by Liu et al. (2022) is not effective for predicting sickness and hence is not used in the current study. The parameters used for the “conflict generation” model in this paper are directly taken from earlier publications on the SVC model (Wada et al., 2020; Liu et al., 2022) and listed in Table 2.

**Table 2:** SVC model parameters.

**Parameter class**	**Parameter symbol**	**Value**	**Explanation**
Anticipation gains	*K*_*a*_	0	Fully passive motion with no anticipation assumed
	*K*_ω_	0	
Vestibular feedback gains	*K*_*ac*_	1	As in Wada et al. (2020)
	*K*_*vc*_	5	
	*K*_ω*c*_	10	
Visual feedback gains	*K*_*gvis*_	0	VV gain set to zero
	*K*_ω*vis*_	10	VR gain as in Wada et al. (2020)
Perception time constants	τ (s)	5	As in Liu et al. (2022)
	τ_*scc*_ (s)	7	

The second part of the model is the “conflict accumulation” model, which accumulates (integrates) the sensory conflict predicted by the first part of the model framework to estimate the build-up of motion sickness over time. The Euclidean norm of the sensory conflict output by the “conflict generation” model is used as the input to the “conflict accumulation” model. Usually, the conflict is integrated using a Hill function combined with a leaky integrator to output a group-level sickness metric such as Motion Sickness Incidence (MSI), which quantifies the percentage of the population that becomes motion sick (Wada et al., 2020; Liu et al., 2022). However, this integration is insufficient to capture the unique dynamics of motion sickness, including hypersensitivity, which is the faster than normal increase of motion sickness after already being subjected to motion sickness before. Thus, we adopt the more advanced model by Oman (1990), which is a five-parameter nonlinear model that integrates the conflict (see the right part in Figure 1), with fast and slow pathways combined with leakage. The fast path has a low time constant (*T*_1_) and models the direct response to a sickening stimulus. The slow path has a high time constant (*T*_2_) and captures the slower secondary effects of sickening stimuli, such as recovery and hypersensitivity. This is also relevant in simulators where a sudden increase in motion incongruence can make participants hypersensitive. This has been observed by Cleij et al. (2018) and Kolff et al. (2022), where it was shown that motion tends to be momentarily bad but not continuously. Both pathways have a gain (*K*_1_ and *K*_2_) to control their contribution. Additionally, there is a power law (*p*) at the model's output to account for nonlinear scaling effects.

### 2.4 Accumulation model: parameter reduction

In addition to studying the accuracy of the Accumulation Model (AM) with all 5 model parameters (*T*_1_, *T*_2_, *K*_1_, *K*_2_, *p*) fitted individually, we consider reduced parameter variations of the AM model to optimize for a “minimum effective” number of individually fitted parameters. Such model reduction is important to enhance the generalisability, efficiency, and interpretability of the proposed personalized accumulation model. For example, in many cases, a reduced number of model parameters improves model generalisability due to a reduced risk of over-fitting. Furthermore, a reduced number of tunable parameters simplifies adjusting the model to different individuals' sickness characteristics and may facilitate a more computationally efficient implementation.

As the main basis for model parameter reduction, in this paper, median measured values and empirical relations between different parameters reported in previous studies are used, e.g.:

- *T*_1_ = 60 s (Oman, 1990)
- *T*_2_ = 7*T*_1_ (Irmak et al., 2020)
- *K*_1_: median value reported for each dataset:
  ° *K*_1_ = 2 for Slalom Drive dataset
  ° *K*_1_ = 18 for Car and Simulator dataset
  ° *K*_1_ = 9 for NDRT Drive dataset
- *K*_2_ = 5*K*_1_ (Oman, 1990)
- *p* = 0.4 (Irmak et al., 2022)

The *K*_1_ values were obtained from the median values estimated with the AM5 model (see Table 3 for details of the AM5 model) on each dataset. It should be noted that the assumed *K*_1_ values are different for each dataset as the motion inputs in each dataset were different. In the “Slalom Drive,” head motion was recorded, but in the “Car and Simulator” and “NDRT Drive,” only vehicle motion was recorded. Hence, a difference in the magnitude of especially the rotations between datasets exists, which explains the difference in estimated *K*_1_ gains. This is shown in Section 1 of Supplementary material where the “Car and Simulator” dataset clearly has the smallest magnitudes of angular velocities. This could also be partly due to the difference in average motion sickness susceptibility of the participants in each experiment; the “NDRT Drive” dataset (with *K*_1_ = 9) could have less motion sickness susceptible participants than the “Car and Simulator” dataset (with *K*_1_ = 18) due to the lower observed median *K*_1_ values.

**Table 3:** Reduced parameter versions of the “conflict accumulation” model (AM).

**Model**	**Number of free parameters**	**Estimated/fixed parameters**				
		*K*_1_	*K*_2_	*T*_1_ **(s)**	*T*_2_ **(s)**	*p*
AM5	5	✓	✓	✓	✓	✓
AM4a	4	✓	5*K*_1_	✓	✓	✓
AM4b	4	✓	✓	✓	7*T*_1_	✓
AM3	3	✓	5*K*_1_	✓	7*T*_1_	✓
AM2	2	✓	5*K*_1_	✓	7*T*_1_	0.4
AM1a	1	2/18/9	5*K*_1_	60	✓	0.4
AM1b	1	2/18/9	✓	60	7*T*_1_	0.4
AM0	0	2/18/9	5*K*_1_	60	7*T*_1_	0.4

Check marks indicate the free model parameters estimated for each individual.

We use combinations of these assumptions to obtain reduced implementations of the AM compared to its original definition, i.e., AM5 (Oman, 1990). Table 3 summarizes the different cases of the accumulation model, indicating which parameters are estimated (marked with a ✓) and which parameters are set based on one of the above assumptions. AM0 is the model with only group-averaged parameters. The *a* and *b* versions of AM1 and AM4 are models with same numbers of parameters (1 for AM1 and 4 for AM4), but with different subsets of parameters being assumed/estimated.

### 2.5 Accumulation model: parameter estimation

To effectively capture individual differences in motion sickness susceptibility with the “conflict accumulation” model, we choose to model the different conditions tested by the same individual in each experiment together, i.e., using a single set of parameters for each individual. This approach enhances generalisability and enables the model to predict motion sickness across a wider range of conditions. This assumes that people respond exactly the same way to conflicts even in very different settings (e.g., internal vs. external vision).

To fit the model parameters, a constrained optimisation problem is defined and solved in *MATLAB* with the *fmincon* solver using the *sqp* algorithm. Furthermore, *multistart* was used to simultaneously find 16 local minima and then select the overall optimum. This enhances the probability of finding the global minimum of the optimisation problem. The Root-Mean-Square Error (RMSE) between the measured and predicted MISC responses, as a function of the parameter vector $\text{x}={\left(T_{1},T_{2},K_{1},K_{2},p\right)}^{T}$, is defined as the cost function for model fitting for each individual. When fitting multiple conditions simultaneously, this means that the cost function is the sum of RMSE values for all *n*_*c*_ conditions within each dataset for each individual. The optimisation problem is mathematically defined in Equation 1, where RMS stands for “Root Mean Square.”


(1) $$\begin{array}{l} \hat{\text{x}}=\underset{\text{x}}{\text{arg min}}\overset{n_{c}}{\underset{i=1}{\sum }}\text{RMS}\left[{\text{MISC}}_{meas,i}-{\text{MISC}}_{pred}\left(\text{x}\right)\right] \end{array}$$

After these models are fitted and their corresponding errors are calculated, we try to select the best model which balances accuracy with loss of generalisability and overfitting. To show this statistically, we used various model selection criteria, such as the Akaike information criterion (AIC) by Akaike (1998) and Bayesian information criterion (BIC) by Schwarz (1978), which take into account the tradeoff between the goodness of fit and the simplicity of the model. In other words, these criteria balance the risk of overfitting and underfitting. A simpler model also means that the simulations will be computationally fast. The model criteria we used are the AIC and BIC, which are defined as,


(2) $$\begin{array}{l} AIC=2k+n\ln\left(RSS/n\right) \end{array}$$

(3) $$\begin{array}{l} BIC=k\ln\left(n\right)+n\ln\left(RSS/n\right) \end{array}$$

where, *k* is the number of parameters, *n* is the sample size and *RSS* is the Residual Sum of Square. The model with the lowest score is selected as optimal. The number of parameters (*k*) and the sample size, which is equal to the number of participants (*n*), are shown in Table 4. The *RSS* of each model is calculated by summing the *RSS* for the fits of each participant and each condition. In the model fittings, RMSE was used as a cost function. RMSE is proportional to *RSS*. RMSE is the square root of the average *RSS* per observation. Thus, the cost function used in the model fitting, RMSE, directly relates to *RSS*, and the model fitting algorithm would also optimize the model criteria such as the AIC and BIC.

**Table 4:** Model selection criterion.

**Model**	**Participants (n)**	**Parameters (k)**	**RMSE**	**mBIC (*c* = 2)**
AM0	44	0	2.06	101.92
AM1a	44	1	1.72	93.49
AM1b	44	1	1.78	96.66
AM2	44	2	1.54	**91.18**
AM3	44	3	1.32	98.19
AM4a	44	4	1.32	92.8
AM4b	44	4	1.32	92.82
AM5	44	5	**1.3**	99.5

Bold numbers show the smallest value in each metric. The total number of participants is 44 (20 from the “Car and Simulator dataset” and 24 from the “NDRT Drive” dataset) for all accumulation models.

However, in applying the AIC and BIC to our problem, the penalty on the number of parameters in both criteria—defined as 2*k* or *k*ln(*n*) in Equations 2, 3, respectively—was found to be insufficient. This insufficiency resulted in the selection of the model with the least error by the criteria. To solve this problem, we followed Drop et al. (2018), where a similar situation was observed. Here, they increased the gain on the term of *k* to increase the penalty. We did the same, and the modified BIC is defined as follows,


(4) $$\begin{array}{l} mBIC=c\;k\ln\left(n\right)+n\ln\left(RSS/n\right) \end{array}$$

where *c* is the model complexity (number of parameters) penalty parameter, which is to be tuned to avoid false positives while maintaining sensitivity to small yet important contributions. To do this, we calculated mBIC values with *c* varying from 1 to 5. We then choose the value of *c* to not select AM0, which is a group-averaged model, or AM5, which is the model with the most number of parameters estimated. With the chosen value of *c*, we find the model with the least mBIC value. This model will be the best the tradeoff between the goodness of fit and the simplicity of the model.

### 2.6 Probabilistic parameter distribution model

To facilitate offline prediction of individual variations in motion sickness development with the proposed model, a statistical model that describes the distribution of the parameters across participants is needed. To estimate such a predictive probabilistic model from our considered datasets, first, the estimated parameter sets for the best model (as per the conditions outlined in Section 2.5) are clustered into three groups using a k-means clustering algorithm. While attempts were made to use more than three clusters, the results consistently showed three prominent clusters, with any additional clusters being very small and closely resembling the original three. These three clusters effectively classify the parameter sets into three distinct groups of motion sickness susceptibility: high, medium and low. Using these clusters, a three-component Gaussian Mixture Model (GMM)—i.e., a weighted sum of three Gaussian distributions—is used to model the distribution of the parameter set. This model is given by:


(5) $$\begin{array}{l} p\left(\text{x}\right)=\overset{3}{\underset{j=1}{\sum }}\pi_{j}N\left(\text{x}|\mu_{j},\Sigma_{j}\right) \end{array}$$

Where:

- **x** is the parameter set
- *p*(**x**) is the probability density function of the GMM.
- π_*j*_ is the weight of the *j*-th Gaussian component, satisfying $\overset{3}{\underset{j=1}{\sum }}\pi_{j}=1$.
- $N\left(\text{x}|\mu_{j},\Sigma_{j}\right)$ is the Gaussian density function, defined as:

(6) $$\begin{array}{l} N\left(\text{x}|\mu_{j},\Sigma_{j}\right)=\frac{1}{2\pi |\Sigma_{j}{|}^{1/2}}\exp\left(-\frac{1}{2}{\left(\text{x}-\mu_{j}\right)}^{⊤}\Sigma_{j}^{-1}\left(\text{x}-\mu_{j}\right)\right) \end{array}$$

In this expression, ***μ***_*j*_ is the mean vector of the *j*-th Gaussian component, and Σ_*j*_ is the *k* × *k* covariance matrix where *k* is the number of parameters in the model. These are fit using *fitgmdist* function in *MATLAB* with the clustering from the k-means algorithm as the starting point.

Finally, using the fitted Gaussian mixture model, we sample 1,000 random parameter sets and use them to predict motion sickness (in MISC) on the “Sickness Recreation” dataset (an unknown dataset to our fitting study). This way, we validate the ability of our model to predict motion sickness in a new scenario.

## 3 Results

This section presents the results of our analysis focused on the proposed new model framework that combines an average “conflict generation” model with an individualized “conflict accumulation” model to capture differences in individual motion sickness susceptibility. In Section 3.1, the model fitting results for various datasets are presented to show the performance of the model with varying numbers of parameters. This outcome is used to demonstrate the effectiveness of our model framework and determine the optimal number of parameters required for the recreation of motion sickness at the individual level. In Section 3.2, the probabilistic model for the observed variation in accumulation model parameters across individuals is extracted from the data and tested on a new population.

### 3.1 Accumulation model performance and parameter selection

First, results are presented for the Slalom Drive dataset by Irmak et al. (2020) demonstrating the performance of the model with varying vision conditions. This is followed by results for the Car and Simulator dataset by Talsma et al. (2023) and NDRT Drive by Metzulat et al. (2024), where the adaptability of the model to real-world driving and driving simulators is shown. Fits of the model framework, with different accumulation models (AM), to the actual MISC responses with Motion Sickness Incidence (MSI) predictions overlayed are shown first, followed by a comparison of the models' RMSE values. It is demonstrated that the use of our model framework with individualization improves the accuracy of the simulation of motion sickness compared to the use of MSI. In addition to this, a parameter study is conducted to find the minimum effective implementation of the model.

#### 3.1.1 Model performance

**Figure 6:**
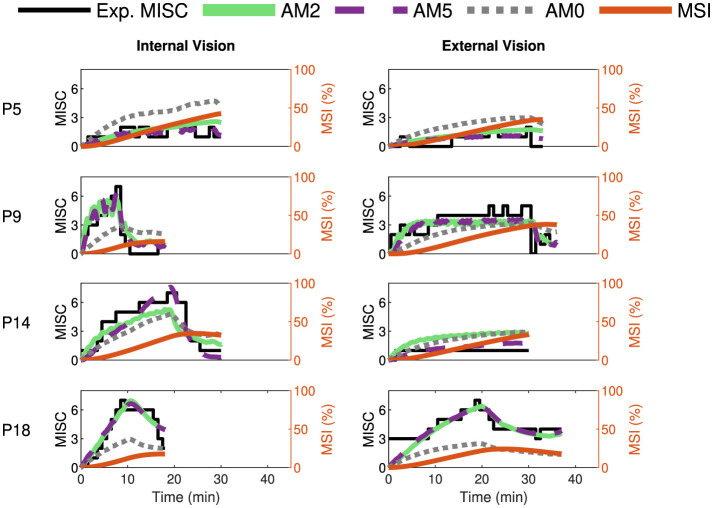
Motion sickness responses (MISC) in Slalom Drive experiments by Irmak et al. (2020) in black, fitted AM2 model predictions (MISC) in green, fitted AM5 model predictions (MISC) in dashed violet, fitted AM0 model predictions (MISC) in dotted gray, and MSI predictions from Hill function in orange for four participants (participant label shown on the left) for the conditions of internal **(left column)** and external **(right column)** vision.

Figure 6 shows the comparison of the most relevant accumulation models with experimental recorded motion sickness responses (MISC) for a representative selection of participants from Irmak et al. (2020)'s experiment for the conditions of internal (left column) and external vision (right column). The obtained model fits for the rest of the participants are shown in Section 2 of Supplementary material. The experimentally reported MISC is shown in black, and MSI predictions (obtained directly using the model from Wada et al., 2020) are shown in orange. The predictions of our proposed combined model are shown for only three out of the eight models in Table 3: AM0 in dotted gray, AM2 in solid green, and AM5 in dashed violet. AM5 is the original version of the accumulation model, with all five parameters being estimated individually. AM0 represents the model with group-averaged parameters. AM2 provides the best compromise fit with only two individual parameters. The AM0 model and MSI predictions both use the same parameter settings for all participants. Any difference in their predictions is thus due to differences in the vehicle motion used as input to these models. In Figure 6, we show MSI for its full range of 0%–100% and MISC across a range of 0–8. These *y*-axis limits are chosen solely to make both sets of data values readable from the graph and enhance their clarity.

It is evident that our approach of estimating parameters for each individual (in particular for AM2 and AM5 models) offers improved accuracy in predicting MISC responses compared to the use of group-averaged parameters (AM0). The average RMSE reduces from 1.94 (AM0) to 1.13 (AM2) and 0.74 (AM5). This proves that using parameters estimated for each individual (AM2 and AM5) is 40% and 60% more accurate, respectively, than using group-averaged parameters, as in the AM0 model. For example, for P5 and P18 in Figure 6, it is clear that AM0 greatly underestimates the measured MISC values.

Another important observation is that all accumulation models are able to capture the recovery from motion sickness. This recovery occurs when the sickening stimuli are stopped, and the participant is allowed to rest. This is more evident for P9 and P14 (second and third row in Figure 6). Consistent with Irmak et al. (2020), the MSI prediction (in orange) cannot capture this reduction in motion sickness.

**Figure 7:**
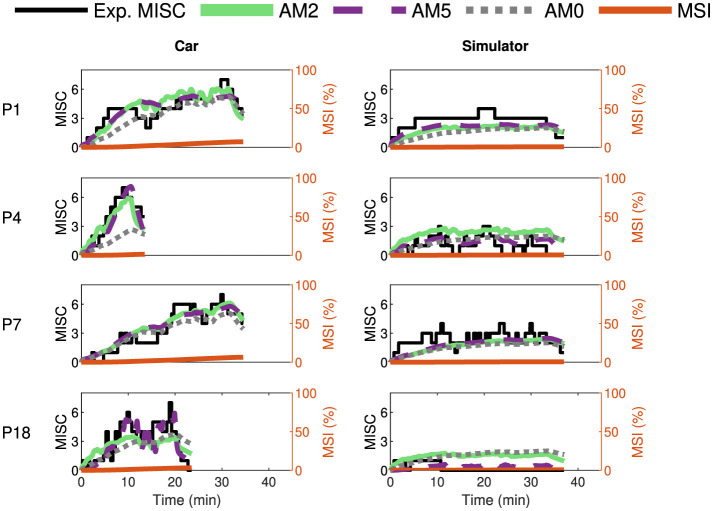
Motion sickness responses (MISC) in Car and Simulator experiments by Talsma et al. (2023) in black, fitted AM2 model predictions (MISC) in green, fitted AM5 model predictions (MISC) in dashed violet, fitted AM0 model predictions (MISC) in dotted gray, and MSI predictions from Hill function in orange for four participants (participant label shown on the left) for the case in the car **(left column)** and the simulator **(right column)**.

**Figure 8:**
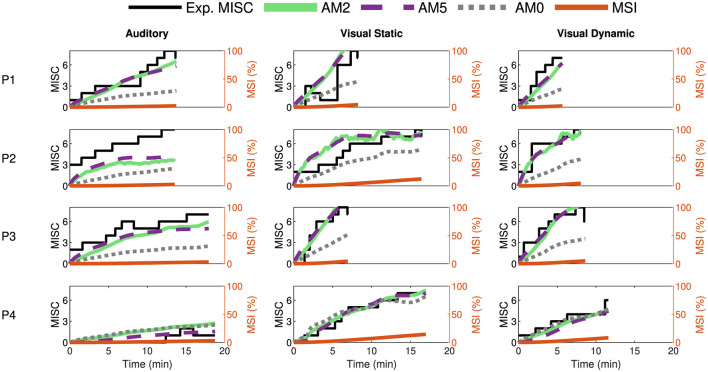
Motion sickness responses (MISC) in NDRT Drive experiments by Metzulat et al. (2024) in black, fitted AM2 model predictions (MISC) in green, fitted AM5 model predictions (MISC) in dashed violet, fitted AM0 model predictions (MISC) in dotted gray, and MSI predictions from Hill function in orange for four participants (participant label shown on the left) for the case in the auditory **(left column)**, visual static **(middle column)** and the visual dynamic **(right column)** task.

Furthermore, we tested the models by fitting them on additional datasets such as the Car and Simulator dataset by Talsma et al. (2023) and the NDRT Drive dataset by Metzulat et al. (2024). The car and simulator dataset had 24 participants, each experiencing motion with external vision in a real-world car and in a simulator. The NDRT Drive experiment had 20 participants, each experiencing three different Non-Driving Related Tasks (NDRT). Figures 7, 8 again show the experimental recorded motion sickness responses (MISC) in black, MSI predictions in orange, and fitted model predictions in green, violet, and gray for four out of the 24 participants for both cases. It can be seen that even with two parameters, the fits of the AM2 model are very close to the actual MISC responses in both datasets. The MSI prediction values are very low, especially in the Car and Simulator dataset. This is due to the low levels of conflict generated in these experiments (0.12 *m*/*s*^2^ mean RMS conflict) compared to other datasets, such as the Slalom Drive, which has 1.15 *m*/*s*^2^ mean RMS conflict. As opposed to the MSI prediction, which is a population-level prediction, the proposed “conflict accumulation” model is able to account for these combinations of low levels of conflict and high susceptibility of the participants. The fits for the rest of the participants are available in Section 2 of Supplementary material.

#### 3.1.2 Model parameter selection

**Figure 9:**
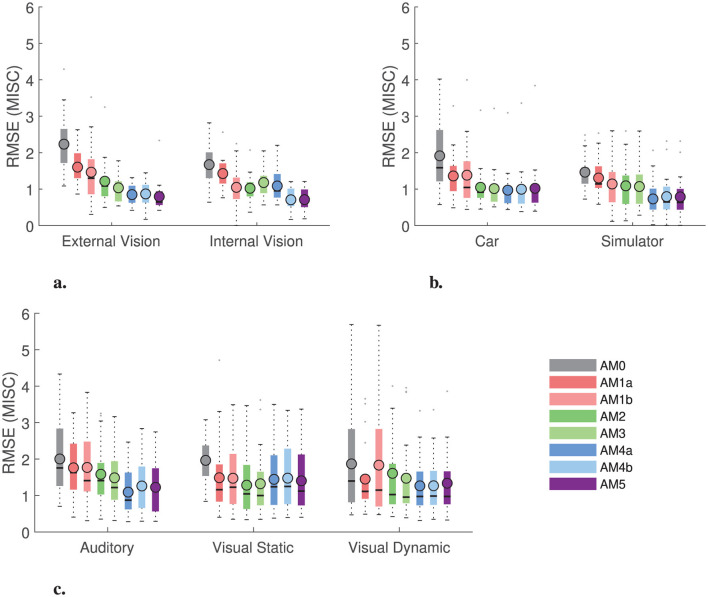
Root mean squared error (RMSE) between the predicted MISC from the models and the actual MISC for the Slalom Drive, Car and Simulator and NDRT Drive datasets. Also shown are the mean (circled), median (horizontal solid line), and interquartile range (in a colored rectangle). **(A)** Slalom drive dataset. **(B)** Car and simulator dataset. **(C)** NDRT Drive dataset.

In the previous section, it was observed that the model with two estimated parameters (AM2) per individual captures the individual responses almost as well as the model for which all five parameters are estimated individually (AM5). To show this quantitatively, we evaluated the need for each of the parameters by comparing all eight models in Table 3. From Figure 9A, it is clear that reducing the number of parameters below two leads to a 36% increase in RMSE (from 1.2 to 1.6 in internal vision case and from 1.0 to 1.4 in external vision case when comparing AM2 to AM1a model) in the Slalom drive dataset. Hence, any model with two or more parameters is sufficiently accurate (with RMSE around 1 MISC) to capture individual motion sickness development. We also compared the individual models with a group-averaged version of the accumulation model (AM0), where the parameters are the same for all participants in the dataset. It is observed that AM0 has, on average, 1.7 times higher RMSE as compared to the AM2 model (for example, from 1.22 to 2.25 in the internal and from 1.04 to 1.63 in the external vision case of the slalom drive dataset). Overall, Figure 9A again shows the improvement obtained with individual model fits compared to using group-averaged parameters.

Furthermore, to demonstrate that the optimal number of accumulation model parameters for our model framework is 2, the previous test was repeated with the Car and Simulator dataset as shown in Figure 9B, where a similar trend is seen with AM2 model offering a good balance between performance and efficiency. When comparing the individual models with a group-averaged version of the accumulation model, it is observed that the group-averaged model, AM0, has 1.64 times more RMSE as compared to the AM2 model (from 1.03 to 1.96 in the internal and from 1.06 to 1.47 in the external vision case). This increase is equivalent to that found for the Slalom Drive dataset (1.7 times increase). This trend was also seen in the NDRT Drive dataset, where the AM2 model offers the best balance between performance and efficiency (see Figure 9C).

It is clear from the results in Figure 9 that this model framework is able to capture multiple conditions with two individually estimated parameters. Overall, the accuracy is better (by around 60% in RMSE) than using a group-averaged model. This is applicable for various vision conditions, as well as different motions from real cars to simulators.

**Figure 10:**
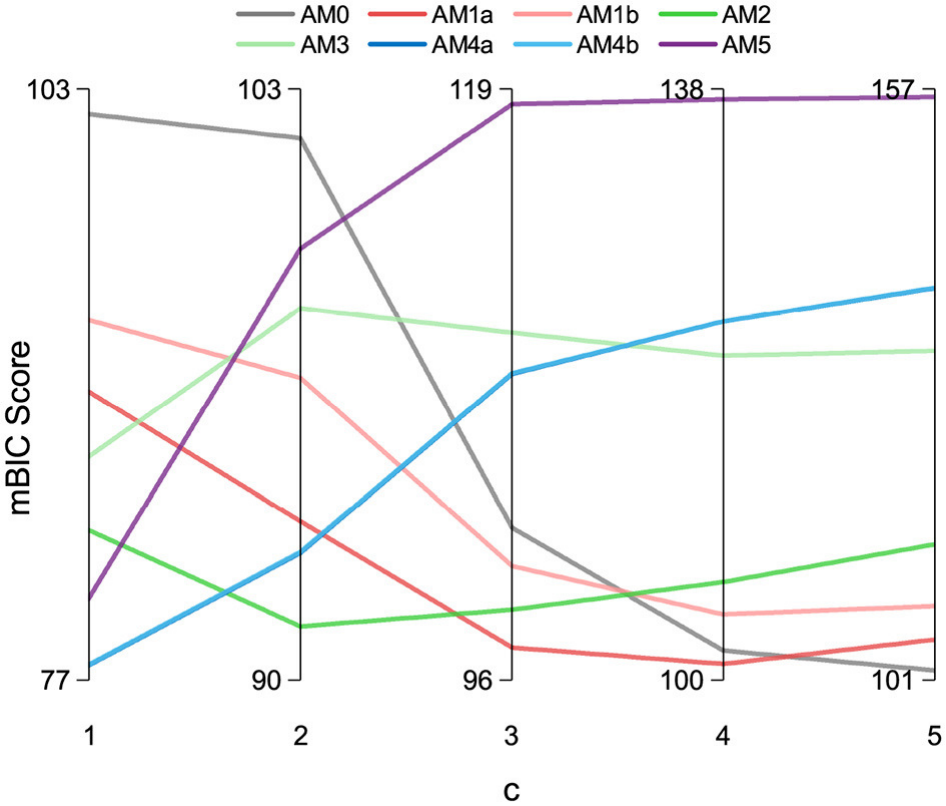
Model selection criterion comparison: mBIC scores with varying value of *c* for various models.

The variation of the mBIC with *c* is plotted in Figure 10. As discussed in Section 2, the selection of the AM0 and AM5 model must be avoided. AM0 model has zero estimated parameters and acts only as an average model. Choosing AM0 would oversimplify and lose generalisability. Similarly, the AM5 model has five estimated parameters, which may cause overfitting even if it fits the data well. When *c* values are 1 and 3, The risk of choosing AM5 and AM0 is high when *c* values are 1 and 3, as these models become more favorable than 50% of other models based on the mBIC metric. With *c* values above 3, the model AM0 becomes very favorable, which is also undesirable. In our fittings, starting with 100 random initial points yields more than 15 solutions when fitting models with more than two parameters (i.e., the AM3, AM4a, AM4b, and AM5 models). In contrast, when using two or fewer parameters (such as the AM2, AM1a, and AM1b models), we achieve two unique parameter solutions, one of which is selected 95 out of the 100 fittings. Out of these three models (AM2, AM1a, and AM1b), the AM2 model demonstrates the highest accuracy with an RMSE of 1.54 MISC. Therefore, to balance accuracy and model complexity, we select (*c* = 2) and opt for AM2 as the optimal model.

Table 4 shows all models with the number of parameters and metrics of their fits—RMSE and mBIC (with *c* = 2). While it is evident from the table that AM5 achieves the lowest RMSE, the mBIC criterion, which incorporates a penalty for the number of parameters, selects AM2 as the optimal model. This highlights the effectiveness of mBIC in balancing model accuracy with parameter simplicity, ensuring a more robust and generalisable model selection. However, it is important to note that the preferred (*c*) parameter may vary with different datasets and applications. Some may prioritize simplicity and opt for a higher value of *c* and choose a single-parameter model instead.

### 3.2 Accumulation model parameter distribution

**Figure 11:**
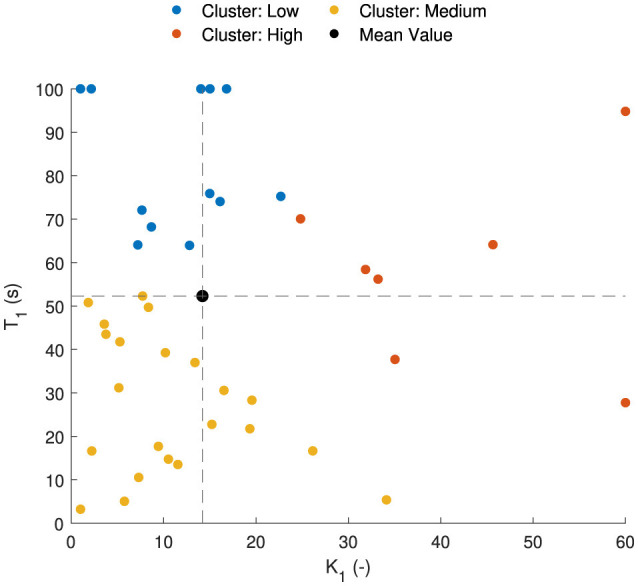
Parameter distribution [estimated gain (*K*_1_) and time constant (*T*_1_)] for the AM2 model. Parameter sets are classified into three groups based on the motion sickness susceptibility—high in red, medium in yellow and low in blue. Black dotted lines show the mean values for *K*_1_ and *T*_1_.

The parameter sets of 44 participants of the Car and Simulator and NDRT Drive datasets are shown in Figure 11 for the AM2 model. Vehicle motion is used as input in both of these datasets. The Slalom drive dataset is omitted here for consistency in parameters due to the slalom drive using head motion instead of vehicle motion, as is the case for the other two. In the figure, it can be seen that there are three distinct sets of parameters, which are found by using the k-means clustering algorithm:

- High susceptibility are those with high *K*_1_ and low *T*_1_, shown as red dots.
- Low susceptibility have the opposite, low *K*_1_ and high *T*_1_, shown as blue dots.
- Medium susceptibility have low *K*_1_ and low *T*_1_, shown as medium dots.

The mean value of the parameters is 15.2 for *K*_1_ and 52.3 seconds for *T*_1_; see black dot in Figure 11. The mean value is located in an area that does not correspond to any individual's parameter values. Therefore, using these mean values to represent a group, which is commonly done, is not correct and does not accurately represent any individual in the population. With this knowledge, representative parameters can be sampled to test motion profiles on different motion sickness susceptibilities. Additionally, percentiles can be defined to see which percentile of subjects do or do not get motion sick.

**Figure 12:**
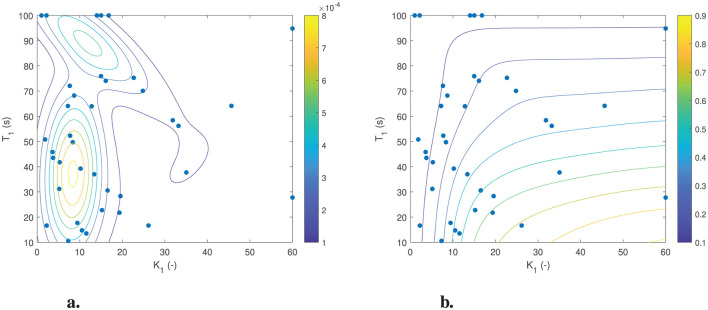
Three component Gaussian mixture model's probability distribution of the parameter sets [estimated gain (*K*_1_) and time constant (*T*_1_)] for the AM2 model. **(A)** Probability density function (PDF). **(B)** Cumulative density function (CDF).

To obtain the probabilistic model, we have fitted a three-component Gaussian mixture model with two dimensions (for the two-parameter AM2 model) on the estimated parameter values obtained for all 44 individuals, see Figure 12. The Figure 12A shows the probability density function of the Gaussian mixture model. This clearly illustrates the three clusters of motion sickness susceptibility identified by the model. Additionally, it highlights the variation in density, indicating that there are more parameter sets associated with medium and low susceptibility compared to those linked to high susceptibility. The Figure 12B, on the other hand, shows the cumulative density function of the Gaussian mixture model. Each colored line represents different percentiles indicating different susceptibility to motion sickness. Any parameter selected along a particular line will yield the same probability of getting motion sickness as any other point on that line. This feature is beneficial for evaluating a motion profile at a designated percentile of the population. Additionally, this representation allows researchers and developers to assess the risk of motion sickness in different scenarios by simply selecting a percentile that matches their target audience. By understanding how motion sickness probabilities vary across different parameters, one can better design experiences or products that minimize discomfort for users. Overall, this approach aids in creating tailored solutions to enhance user comfort and experience in motion-related activities. The corresponding parameters for the Gaussian mixture model can be found in Section 3 of Supplementary material.

**Figure 13:**
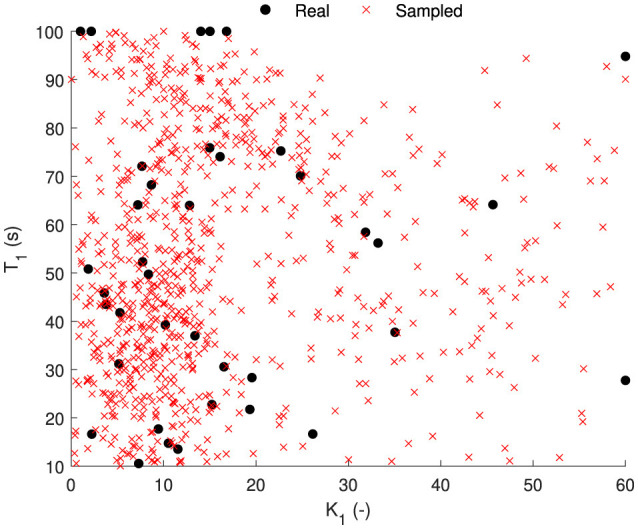
Sampled parameter sets (red cross) from the probability density function of the parameter distribution [estimated gain (*K*_1_) and time constant (*T*_1_)] for the AM2 model (black dots).

With this probabilistic model, we can sample parameters randomly or based on the probability of getting sick (from 0 to 1). Here, Figure 13 shows an example of randomly sampled 1,000 parameter sets overlayed on the actual parameters estimated from the other datasets. The sampled parameters have the same distribution as the actual real estimated parameter sets.

**Figure 14:**
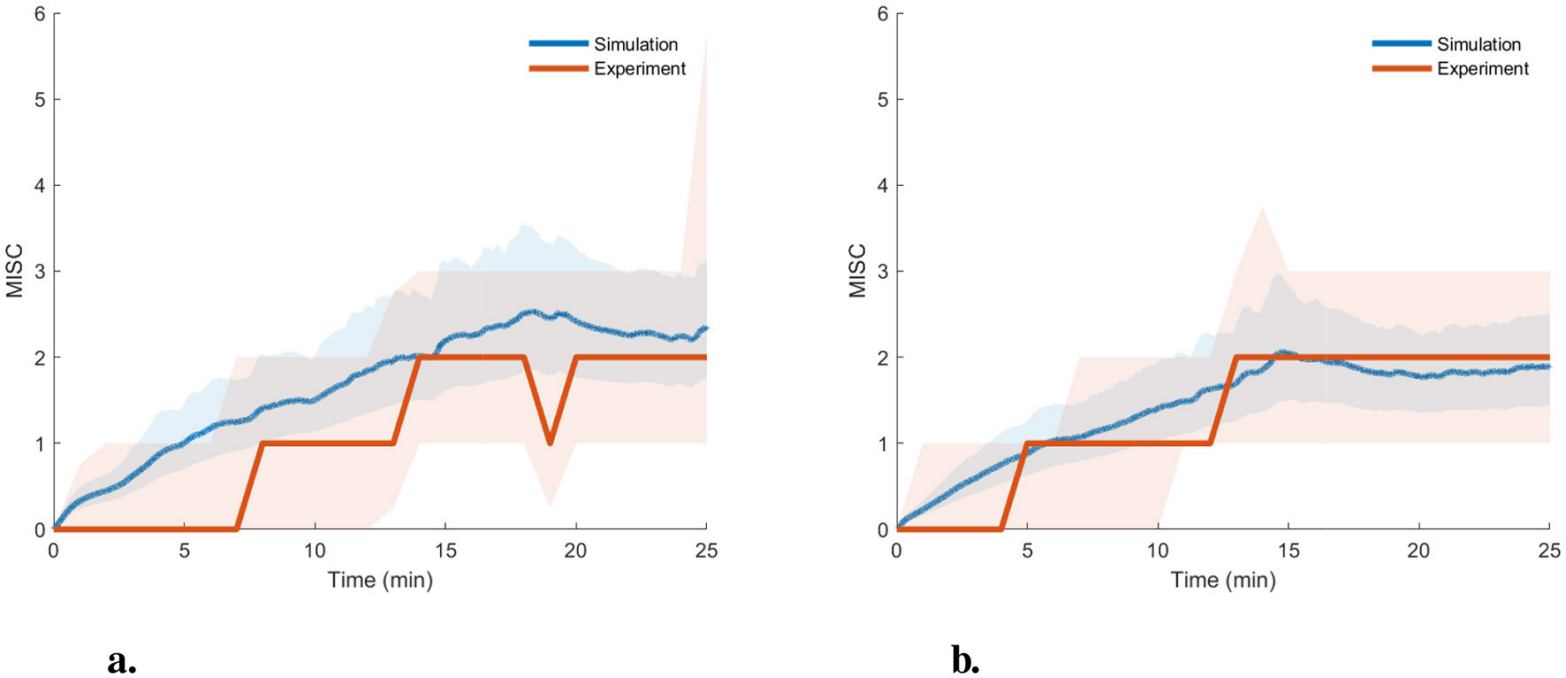
Experimentally reported MISC and predictions of MISC on the “Sickness recreation” dataset (Harmankaya et al., 2024) from sampled parameter sets from the probabilistic parameter distribution model [estimated gain (*K*_1_) and time constant (*T*_1_)] for the AM2 model with the shaded region showing the 25th to 75th percentiles. **(A)** On road. **(B)** On track.

To show a use case of this probabilistic model, we simulated these 1,000 sampled parameter sets on the completely independent Sickness Recreation dataset with 47 participants, see Table 1. This dataset is collected with the same participants being driven manually in a semi-urban environment and in automated mode on a test track with the same vehicle. The median MISC with the 25th and 75th percentile is shown below in Figure 14. This is overlayed with the median and 25th/75th percentiles from the 1,000 sampled parameter sets obtained from the probabilistic model. It is observed that the two MISC traces are highly similar, with an average RMSE of 0.47 (RMSE of 0.65 for “On Road” and 0.29 for “On Track”). However, the model prediction somewhat underestimates the sickness variance. This can be seen especially at the start, where the experiment data shows some people jumping to MISC of 1 very quickly, which is not seen in the model predictions.

Overall, these results indicate that we can use the probabilistic model to predict the variation in expected motion sickness levels for a new population in untested experiments and scenarios, including those using various motion planning or motion cueing algorithms.

## 4 Discussion

### 4.1 Combined model framework for individual motion sickness predictions

This paper introduces and validates a novel combination of models to create a framework to predict an individual's motion sickness level in vehicles and simulators. This model framework combines a group-average “conflict generation” model with an individual “conflict accumulation” model to capture individual susceptibility differences. Furthermore, by using a “conflict generation” model that includes visual inputs, various vision conditions (such as external, internal, and only vision) can be simulated. This is crucial in simulators where motion sickness occurs due to a strong influence of visual cues. We hypothesized that using a “conflict accumulation” model with individualized parameters will result in greater accuracy compared to a model that uses group-averaged parameters. Hence, in this paper, we assessed the feasibility and accuracy of this new model approach for motion sickness predictions.

It is clear from the obtained results (see Figures 6–9, Table 4) that “conflict accumulation” models with individualized parameters enable improved modeling of the motion sickness responses of individuals as compared to using the group-averaged models, as considered for AM0. In addition to this, the “conflict accumulation” models we use (AM0-5) also capture the recovery phase of the experiment (see Figures 6–8) and, theoretically, the hypersensitivity in a following second motion exposure as shown in Irmak et al. (2020, 2022). This recovery phase takes a few minutes and is not captured by often-used MSI predictions that typically implement a leaky integrator with a time constant of 12 min for conflict accumulation (Kamiji et al., 2007; Liu et al., 2022). These results are in line with the work by Irmak et al. (2020) where individualized fits with the same accumulation model (AM4a) reduced the prediction error by a factor of 2 in a slalom drive with a frequency of 0.2 Hz and lateral accelerations with a peak amplitude of 0.4 g with eyes closed. The limitation of this previous work was that no “conflict generation” model was considered; instead, the one-dimensional lateral acceleration conflict was simply used directly as the input to the accumulation model. Our work extends this by using a 6 degrees-of-freedom “conflict generation” model to generate the conflict in realistic driving conditions with varying vision conditions, thereby expanding the coverage to full 6 degrees-of-freedom vehicle motion. Including an explicit “conflict generation model” allows the model to be used in various conditions as well, be it variation in vision or motion.

This way, a single set of parameters, estimated using data from all required conditions, can characterize an individual across various motion and vision conditions. Estimating the parameters on all available conditions is important as it facilitates a more robust modeling of how different factors contribute to motion sickness. It prevents overfitting, as the model is not solely fitted to specific scenarios but is rather modeled by the collection of individual responses across various contexts. This generalisability is crucial in real-world applications, where individuals may encounter unfamiliar situations that were not part of the initial training dataset.

This approach is not limited to the SVC model, which we used as the “conflict generation” model. Any other “conflict generation” model can be used, such as any of the versions of SVC by Kamiji et al. (2007), Liu et al. (2022), and Wada et al. (2020) or the Multi-Sensory Observer Model by Newman (2009) and Clark et al. (2019).

### 4.2 Accumulation model parameter reduction

We investigated reducing the number of estimated individual model parameters. The “minimum effective” model (AM2) well-captured sickness in individuals. While models such as AM5, AM4a, and AM4b provide better accuracy than the AM2 model, they require more estimated parameters (5, 4, and 4, respectively). This higher number of individually estimated parameters may cause overfitting and hinder generalisability, leading to non-unique parameters that reduce model's reliability. By using the relations and values mentioned in previous studies (Oman, 1990; Irmak et al., 2020, 2022), the number of estimated individual parameters could be limited to 2, ensuring uniqueness and avoiding overfitting on the sparse (in conditions) dataset. Overfitting may degrade a real-world performance where conditions differ from training data. A concise and relevant parameter set enhances the robustness and generalisability of predictions, ensuring application in untested situations. This balance between simplicity and effectiveness positions our model as a powerful tool for predicting motion sickness across diverse situations, improving interventions and user experiences. Additionally, a significant reduction in computation time is achieved for the parameter estimation—by a factor of 4 from 48 to 11 s for 40 min of simulation—highlighting the efficiency of the AM2 model. An even larger benefit would be observed in stochastic modeling, where a 5-parameter model would take exponentially more time. However, there is no difference when using these models for online prediction of sickness levels.

### 4.3 Probability distribution of individual parameters

We created a distribution of the parameter sets, which can be used to sample any number of parameter sets (Figure 13) and simulate the motion profile to predict the distribution of motion sickness levels. This has been demonstrated in Figure 14 where the simulated sickness predictions closely match the experimentally observed median sickness levels. The difference in the spread of sickness is likely due to the selection of participants with extremely high or low sickness susceptibility, which is sometimes not correctly reported in the self-reported Motion Sickness Susceptibility Questionnaire. Additionally, at the start of the experiments, some of the participants quickly reported a MISC of 1. Our models do not capture this, but instead, due to the inherent nature of the models, there is a continuous increase in MISC over time. If this is instead discretised as is reported by the participants, the sickness predictions match even better, and the spread of MISC is completely inside the experimentally reported MISC (see Section 4 of Supplementary material). This proves that we can use this probability distribution to predict expected motion sickness levels for untested experiments and scenarios, including those using various motion planning or motion cueing algorithms. Also, this can also be used as a proxy for Motion Sickness Dose Value (MSDV), which is an ISO standard (International Organization For Standardization, 1997) and uses weighted root mean squared acceleration as a measure of motion sickness severity. This has been known to not accurately capture the dynamic nature of motion sickness, especially the quick recovery from sickness, which is not captured by MSDV due to its monotonous nature and lack of any leakage term (International Organization For Standardization, 1997).

### 4.4 Practical applications

The probabilistic approach enables the model to predict variations in motion sickness outcomes across a population, facilitating advanced vehicle motion planning, such as in Li and Hu (2021), and motion cueing algorithms in driving simulators, such as in Jain et al. (2023), and flight simulators, such as in Lewkowicz (2019). Using a model-based control method, this model framework can be included in the plant model to forecast motion sickness levels. This way, motion sickness levels can be controlled by considering each individual's susceptibility. Vehicle motion in automated vehicles and platform motion and tilt coordination in simulators can be optimized to optimize their effect in eliciting motion sickness. Using these models, the motion profiles can be tuned to reduce the dropout of participants in simulator experiments due to motion sickness.

Moreover, this model framework can also be used during the process of experiment design. Algorithms can be benchmarked on different thresholds of motion sickness susceptibility. Offline analyses can be run on a large synthetic sample of a population before real-world testing on humans. This will greatly speed up the testing process.

Thus, our next steps involve applying this model both in pre-experiment and real-time during the experiment.

### 4.5 Challenges and limitations

We now tuned individual parameters of the accumulation model while keeping the (many) parameters of the sensory conflict model constant. This assumes that the conflict generated is the same for all individuals, and the difference in motion sickness development is purely due to the difference in the accumulation of the conflict. This implies that people respond exactly the same way to conflicts, even in very different settings (e.g., internal vs. external vision). This assumption is made because the initial stage of conflict generation primarily involves basic sensory mechanisms all humans share. However, the degree to which different individuals accumulate and tolerate this conflict can vary widely. This variation can be influenced by genetic and psychological factors. Previous experiences and conditioning can also influence susceptibility. Additionally, individuals who frequently experience motion sickness may anticipate symptoms more anxiously, exacerbating the response. However, this assumption contradicts the observations by Irmak et al. (2021), where they found a correlation of 0.74 between an individual's sickness susceptibility and their subjective vertical time constant, which is a parameter in the conflict generation model. This correlation suggests that the subjective vertical time constant plays a significant role in how conflicts are generated and perceived, challenging the notion that conflict generation is uniform across individuals. Consequently, it is highlighted that there is a need for a model that incorporates individual differences in both conflict generation and accumulation in order to more accurately reflect the complex interplay of factors influencing responses to motion sickness.

A main limitation of the model during individual fitting is that it does not fit participants who get highly motion sick with internal vision and do not get sick with external vision well (for example, P14 in Figure 6 and P2 in Figure 8). This sharp shift in motion sickness dynamics cannot be captured by our model framework. This may be due to the inherent difference in conflict generation, which we assume to be the same in all individuals, or to our assumption that gains in the SVC model are independent of the visual condition. Also, in the “NDRT Drive” dataset, there are conditions—“Visual Static” and “Visual Dynamic”—with internal vision that feature varying levels of visual stimuli. We have chosen to model these identically due to the unavailability of control over the effect of vision on conflict generation, as the vision is either represented as 1 or 0. One solution is to model each condition with a separate set of parameters. Doing this, the RMSE was reduced, on average by 35%, from 1.54 to 1 MISC, compared to fitting all conditions together. However, this comes with a significant reduction in predictive accuracy on other conditions by increasing RMSE by 69% from 1.54 to 2.6 MISC. This means we lose any generalisability in modeling other conditions and overfit the model to that specific condition. A better solution would be to estimate at least one additional parameter, one of which could be the vision gain in the “conflict generation” model or other perception parameters. People not only differ in motion sickness susceptibility but also in their perception of vision. Each human will have a different contribution of visual and vestibular signals for their state estimation. By adjusting the vision gain in the “conflict generation” model, the contribution of vision to the estimates can be tuned.

Another limitation is that the model is sensitive to the location of the Inertial Measurement Unit (IMU), i.e., the input of the model needs to be based on either vehicle motion or head motion for it to predict motion sickness reliably. For the modeling of the distribution of parameters, the inputs are vehicle motion. This is due to the larger number of datasets available with vehicle motion measured as compared to datasets with head motion measured. For example, if we take head motion as input, the estimated gain (*K*_1_) is much smaller than for the datasets which use vehicle motion (see Section 5 of Supplementary material). Also, the Hill function accumulation model predicted a reasonable MSI magnitude in the Slalom Drive but highly underestimated sickness in the Car and Simulator dataset. Ideally, we would like to always use recorded head motion, which is more representative of the motion experienced by the vestibular system. If this is not available, we could use biomechanical human/seat models or linear transfer functions to convert six degrees-of-freedom vehicle motion to 6 degrees-of-freedom head motion (Desai et al., 2023; Happee et al., 2023). This way, even when head motion is not recorded, a head motion based stochastic sickness generation model can be used.

## 5 Conclusion

This study presents a novel framework for predicting motion sickness (in MISC) accumulation in time by integrating a group-average “conflict generation” model with an individualized “conflict accumulation” model. By utilizing acceleration and angular rotational data, the model adjusts parameters specific to each individual's motion sickness response, as measured by the Misery Scale (MISC). By simultaneously fitting for various conditions across different datasets, the model successfully estimates a single set of parameters applicable to each participant, offering a highly personalized approach to understanding motion sickness dynamics. A reduction of estimated parameters not only simplifies the model but also optimizes the risk of overfitting, ensuring robust application in real-world scenarios. This framework achieves an average RMSE of 1.54 with just two estimated parameters—a gain (*K*_1_) and a time constant (*T*_1_). The integration of the two models demonstrates significant improvements in predicting motion sickness, achieving better fits by 34% compared to traditional group-averaged models (1.54 RMSE for AM2 vs. 2.06 RMSE for AM0).

Moreover, the modeling of the probabilistic distribution of estimated accumulation parameters enables effective sampling of parameter sets, facilitating predictions for untested scenarios and improving the adaptability of motion sickness assessments. Such flexibility reduces reliance on extensive human testing experiments and accelerates testing processes.

Overall, this research paves the way for more refined and personalized applications in both driving simulators and real-world automated vehicle contexts, promising improved user experiences and outcomes.

## Data Availability

The original contributions presented in the study are included in the article/Supplementary material, further inquiries can be directed to the corresponding author.
