# Supplementary material for: Personalizing motion sickness models: estimation and statistical modeling of individual-specific parameters
Source: Front Syst Neurosci. 2025 Jun 16;19:1531795. doi: 10.3389/fnsys.2025.1531795 (PMC12206751; doi:10.3389/fnsys.2025.1531795)
Supplement: Supplementary file 1 [file Data_Sheet_1.pdf]

Supplementary Material

1 MOTION STIMULI IN EACH OF THE DATSETS

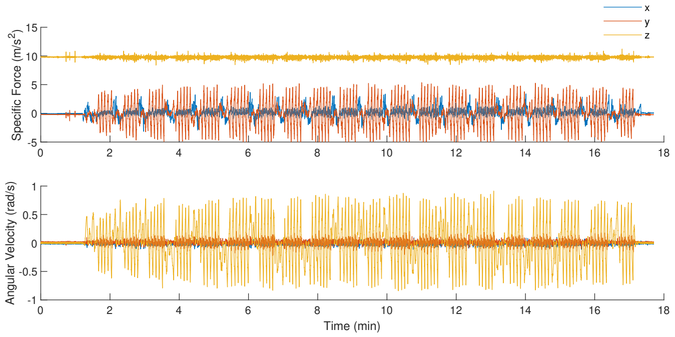

Figure S1. Slalom Drive dataset (Irmak et al., 2020) head specific forces and angular velocities for one participant

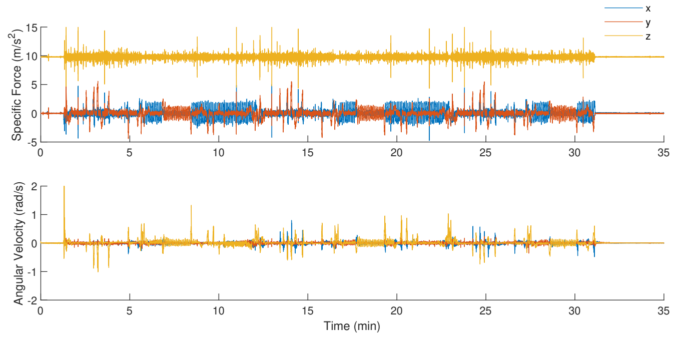

Figure S2. Car and Simulator dataset (Talsma et al., 2023) vehicle specific forces and angular velocities for one participant

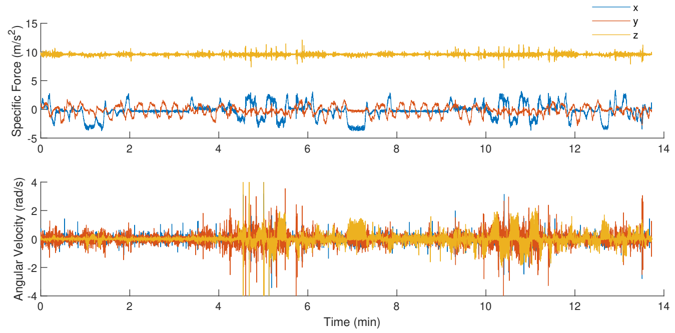

Figure S3. NDRT Drive dataset (Metzulat et al., 2024) vehicle specific forces and angular velocities for one participant

2 FITTING RESULTS FOR ALL DATA

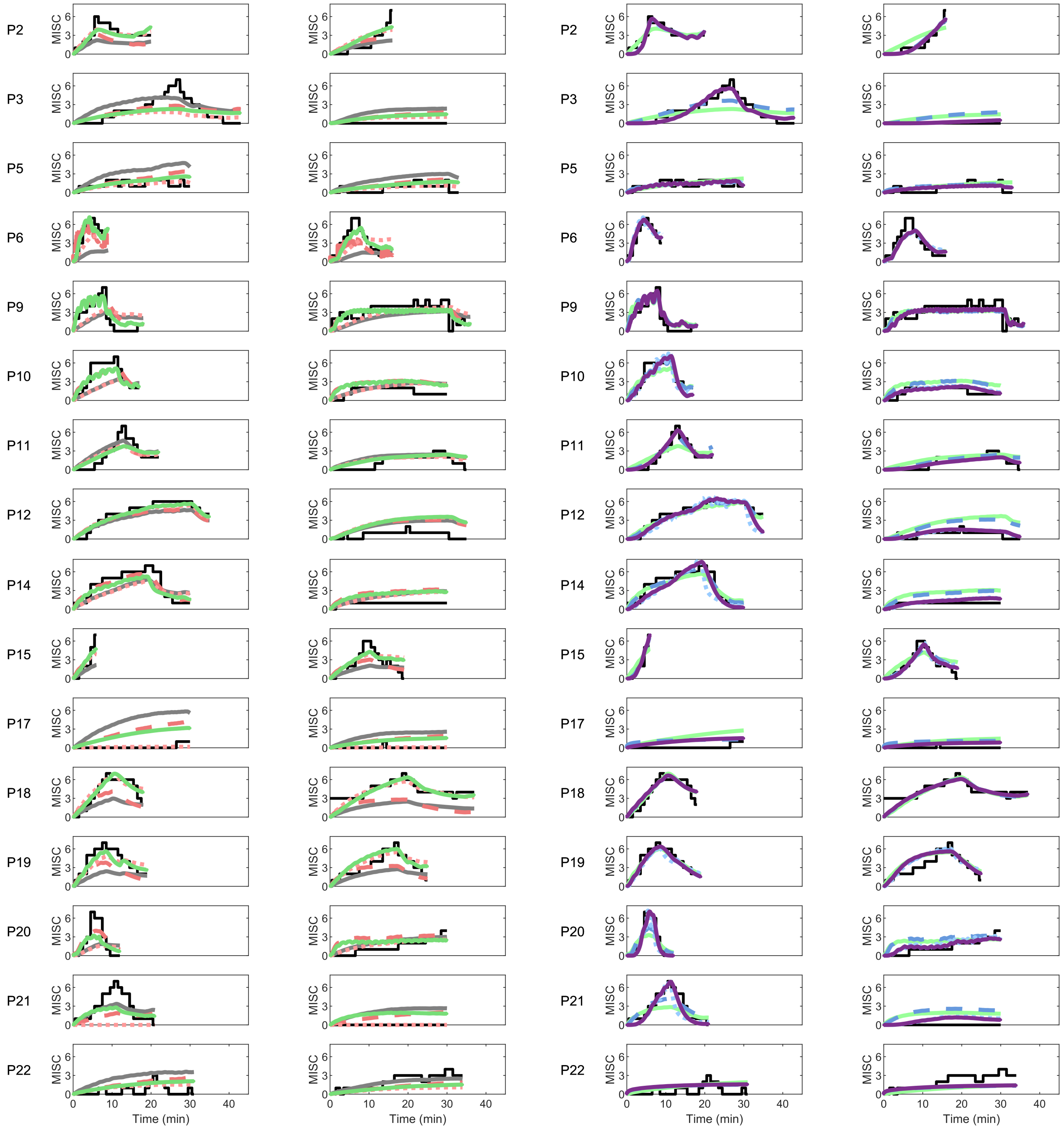

Figure 4a. AM0, AM1a, Am1b, AM2

Figure 4b. AM3, AM4a, Am4b, AM5

Figure 4. Motion sickness responses (MISC) in Slalom Drive by Irmak et al. (2020); experiment in black, fitted AM0 in grey, AM1a in dashed red, AM1b in dotted light red, and AM2 in green, AM3 in light green, AM4a in dashed blue, AM4b in dotted light blue, and AM5 in violet for all participants (participant label shown on the left) for the conditions of internal (left column) and external (right column) vision.

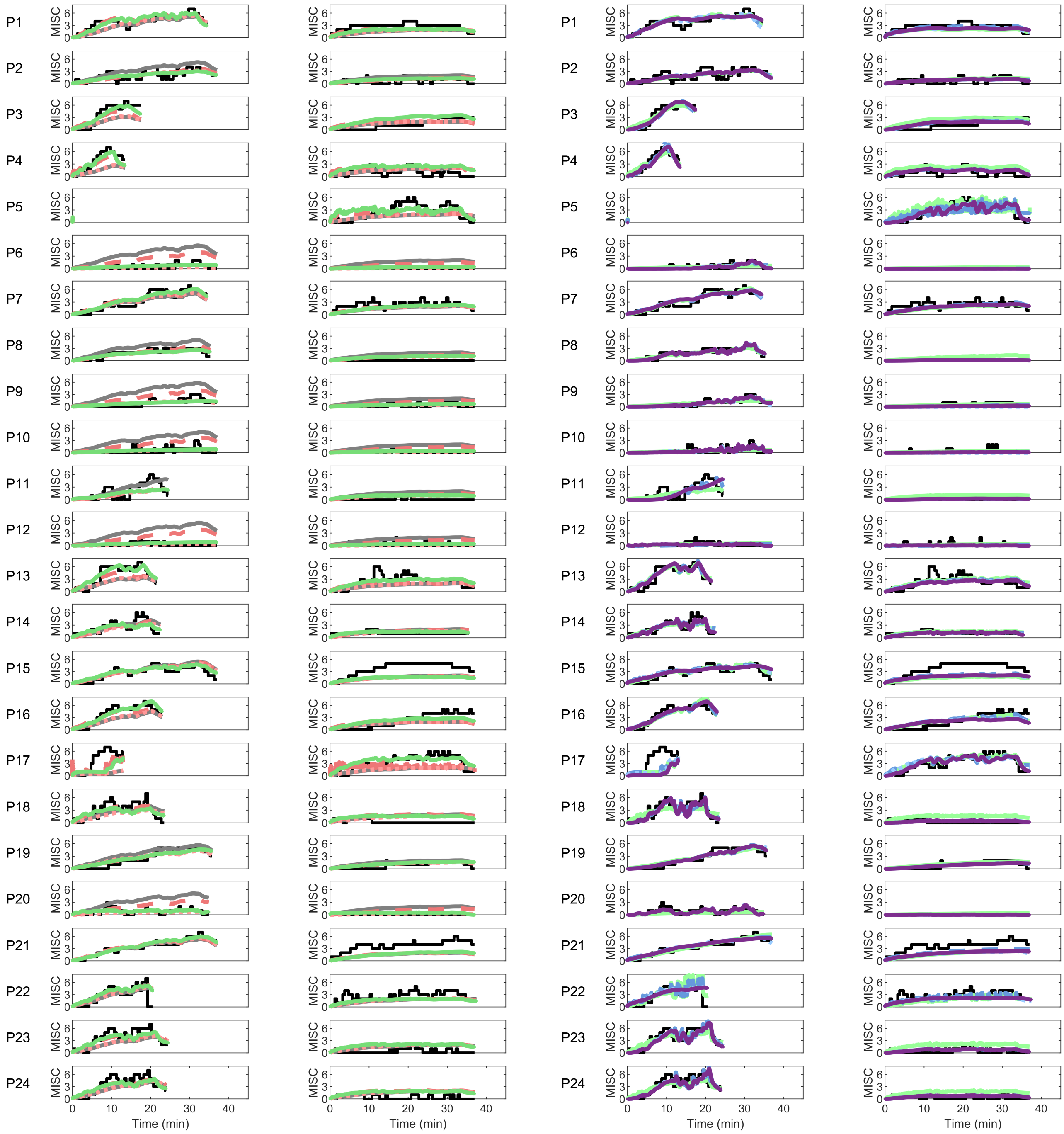

Figure 5a. AM0, AM1a, Am1b, AM2

Figure 5b. AM3, AM4a, Am4b, AM5

Figure 5. Motion sickness responses (MISC) in Car and Simulator by Talsma et al. (2023); experiment in black, fitted AM0 in grey, AM1a in dashed red, AM1b in dotted light red, and AM2 in green, AM3 in light green, AM4a in dashed blue, AM4b in dotted light blue, and AM5 in violet for all participants (participant label shown on the left) for the conditions of internal (left column) and external (right column) vision.

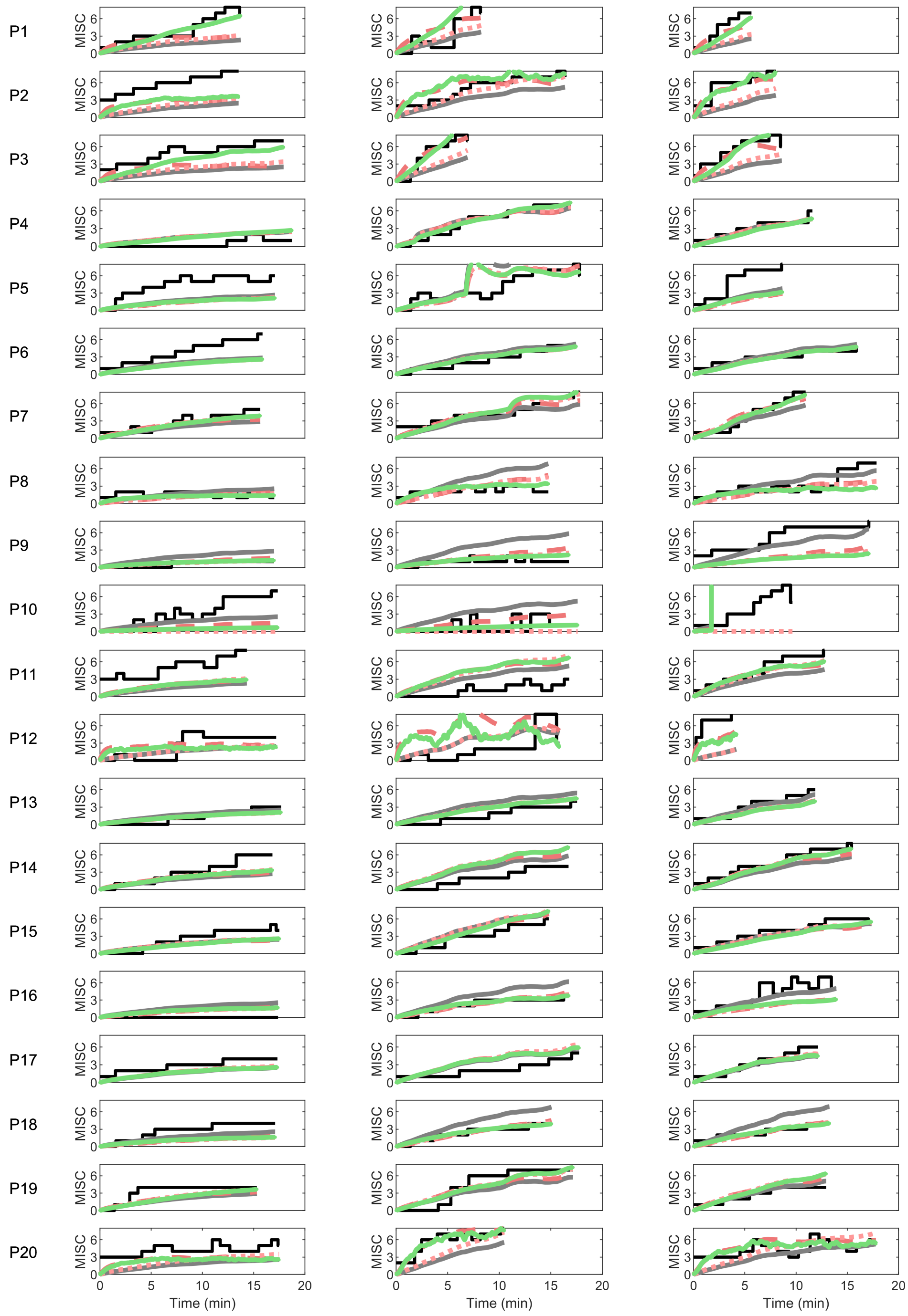

Figure 6a. AM0, AM1a, Am1b, AM2

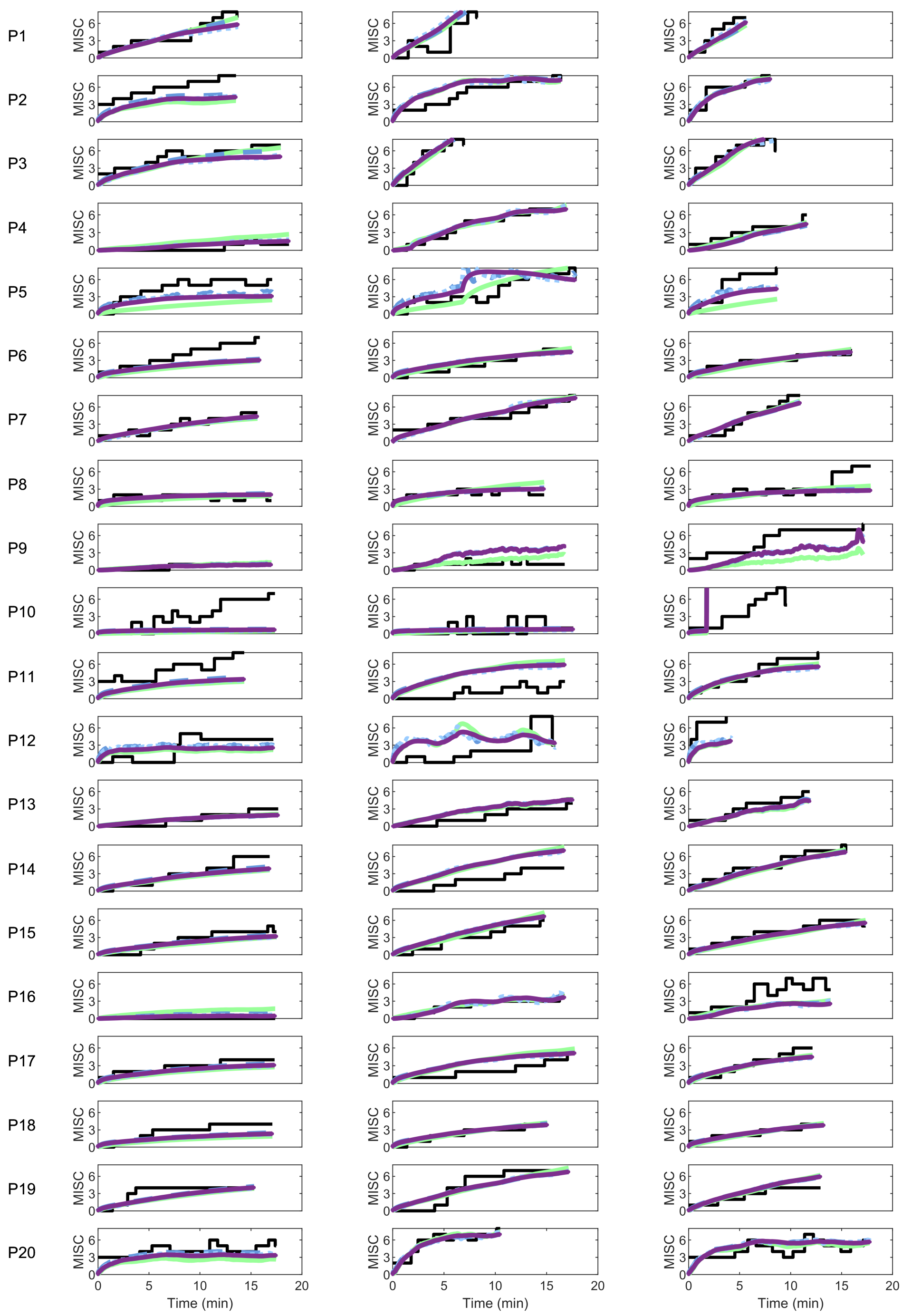

Figure 6b. AM3, AM4a, Am4b, AM5

Figure 6. Motion sickness responses (MISC) in NDRT Drive by Metzulat et al. (2024); experiment in black, fitted AM0 in grey, AM1a in dashed red, AM1b in dotted light red, and AM2 in green, AM3 in light green, AM4a in dashed blue, AM4b in dotted light blue, and AM5 in violet for all participants (participant label shown on the left) for the conditions of internal (left column) and external (right column) vision.

3 PARAMETER DISTRIBUTION MODEL

A 3-component Gaussian Mixture Model (GMM) in 2 dimensions can be expressed mathematically as a weighted sum of three Gaussian distributions. The model is given by:

$$p(\mathbf{x}) = \sum_{j=1}^{3} \pi_j \mathcal{N}(\mathbf{x}|\boldsymbol{\mu}_j, \boldsymbol{\Sigma}_j)$$

Where:

- $p(\mathbf{x})$ is the probability density function of the GMM. - π_j is the weight of the j-th Gaussian component, satisfying $\sum_{j=1}^{3} \pi_j = 1$. - $\mathcal{N}(\mathbf{x}|\boldsymbol{\mu}_j, \boldsymbol{\Sigma}_j)$ is the Gaussian density function, defined as:

$$\mathcal{N}(\mathbf{x}|\boldsymbol{\mu}_j, \boldsymbol{\Sigma}_j) = \frac{1}{2\pi|\boldsymbol{\Sigma}_j|^{1/2}} \exp\left(-\frac{1}{2}(\mathbf{x}-\boldsymbol{\mu}_j)^\top \boldsymbol{\Sigma}_j^{-1}(\mathbf{x}-\boldsymbol{\mu}_j)\right)$$

In this expression, $\boldsymbol{\mu}_j$ is the mean vector of the j-th Gaussian component, and $\boldsymbol{\Sigma}_j$ is the 2x2 covariance matrix.

4 DISCRETISED PREDICTIONS

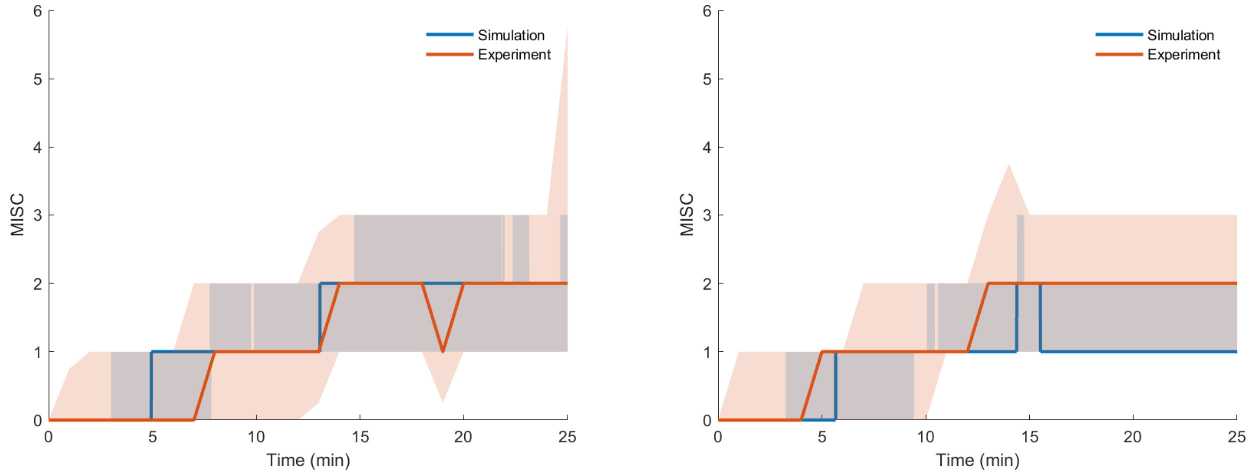

Figure 6a. On Road

Figure 6b. On Track

Figure 6. Experimentally reported MISC and discretised Predictions of MISC on the ‘Sickness recreation’ dataset (Harmankaya et al., 2024) from sampled parameter sets from the probability density function of the parameter distribution (estimated gain (K_1) and time constant (T_1)) for the AM2 model.

5 PARAMETER DISTRIBUTION

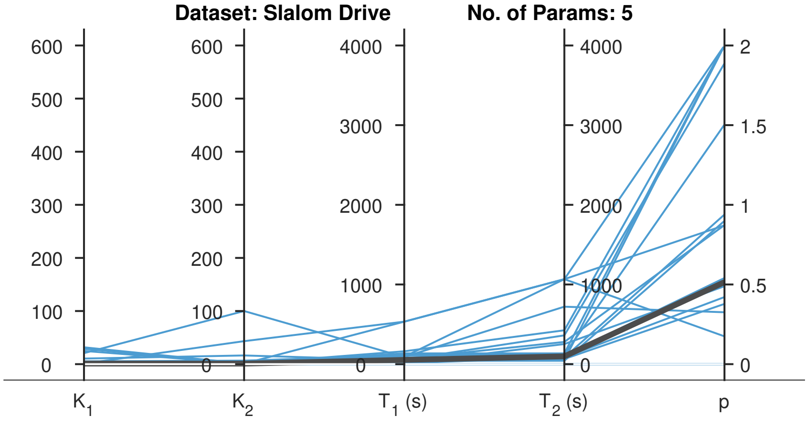

Figure 7a. Slalom drive dataset

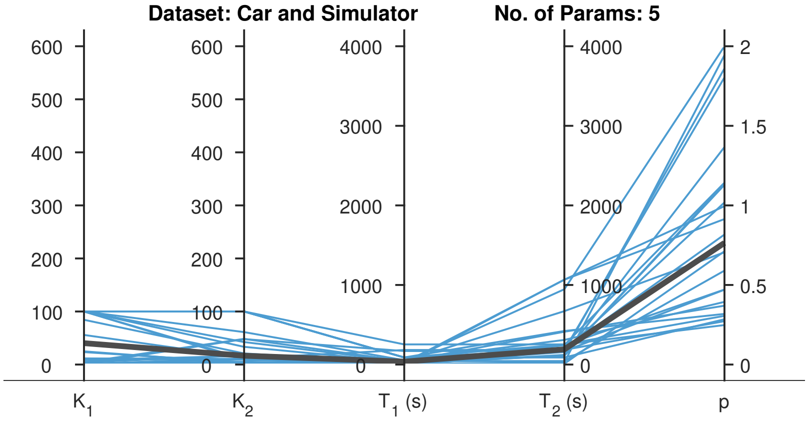

Figure 7b. Car and Simulator dataset

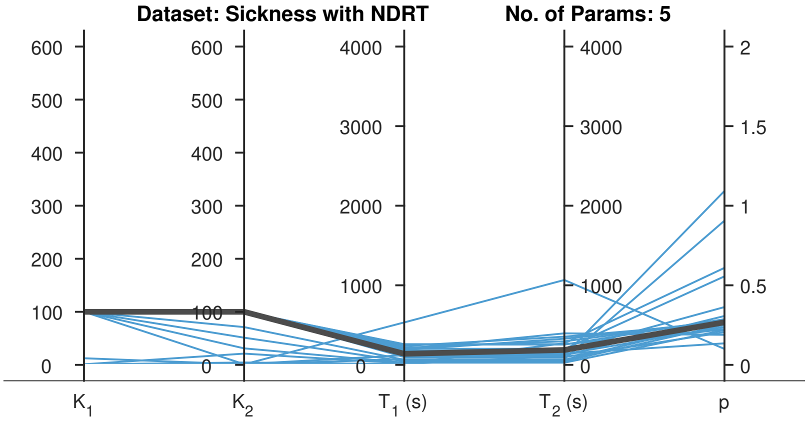

Figure 7c. NDRT Drive dataset

Figure 7. Parameter distribution (estimated gain (K_1) and time constant (T_1)) for the AM5 model (blue) with median values (black) for the three datasets

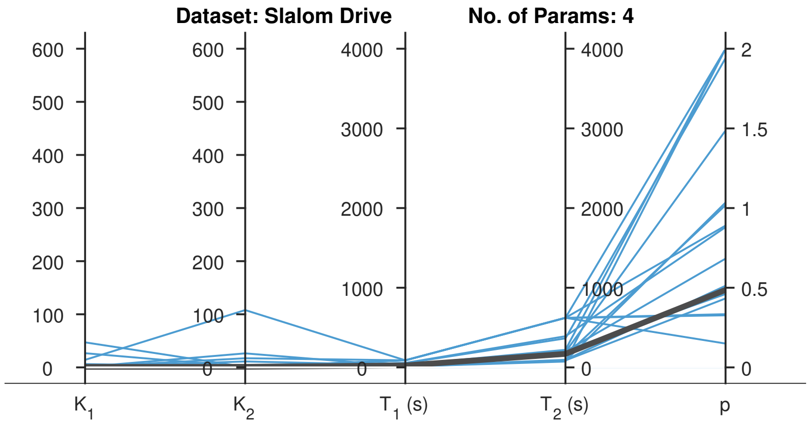

Figure 8a. Slalom drive dataset

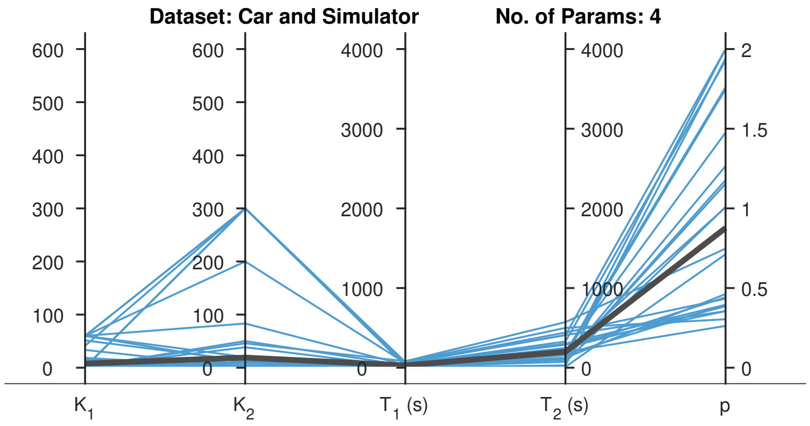

Figure 8b. Car and Simulator dataset

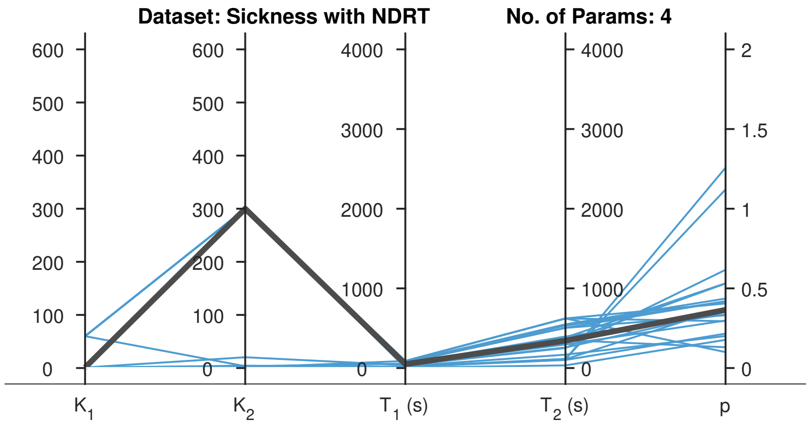

Figure 8c. NDRT Drive dataset

Figure 8. Parameter distribution (estimated gain (K_1) and time constant (T_1)) for the AM4b model (blue) with median values (black) for the three datasets

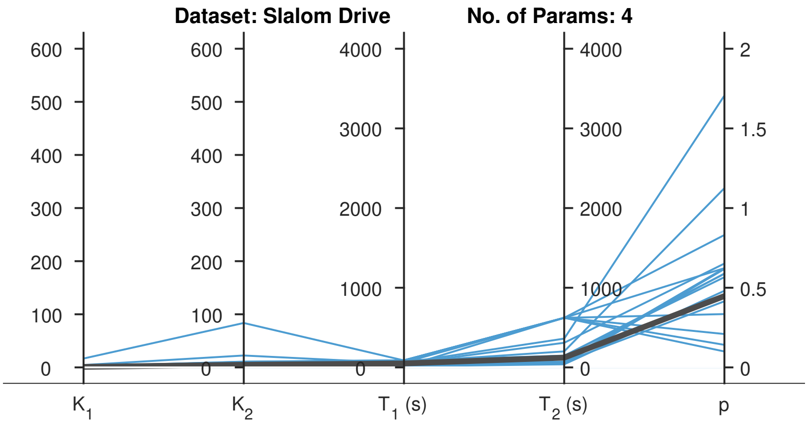

Figure 9a. Slalom drive dataset

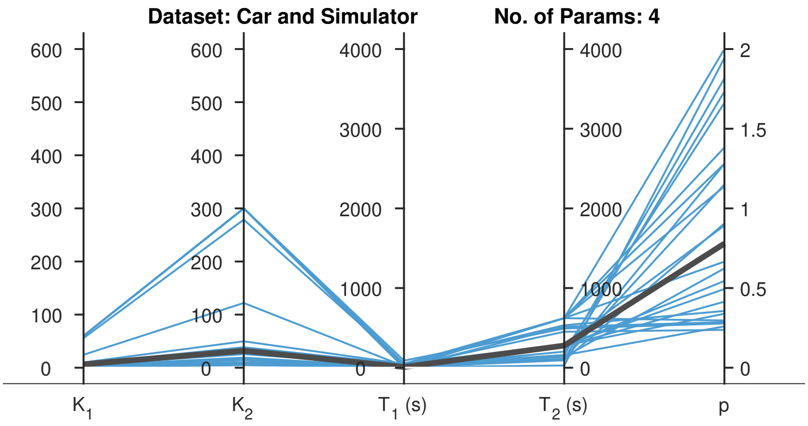

Figure 9b. Car and Simulator dataset

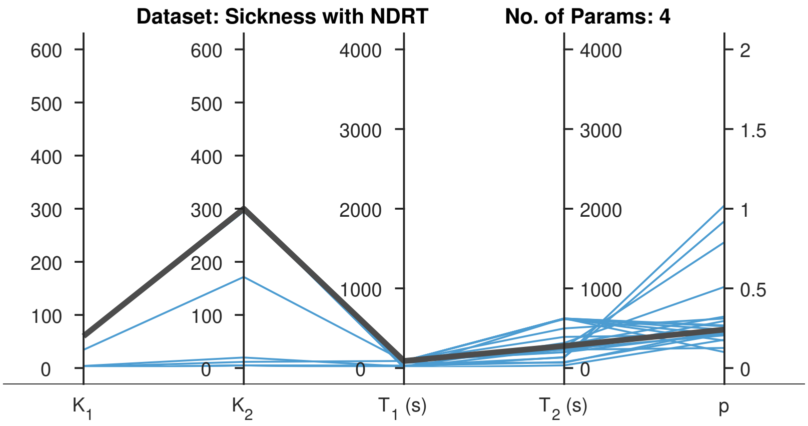

Figure 9c. NDRT Drive dataset

Figure 9. Parameter distribution (estimated gain (K_1) and time constant (T_1)) for the AM4a model (blue) with median values (black) for the three datasets

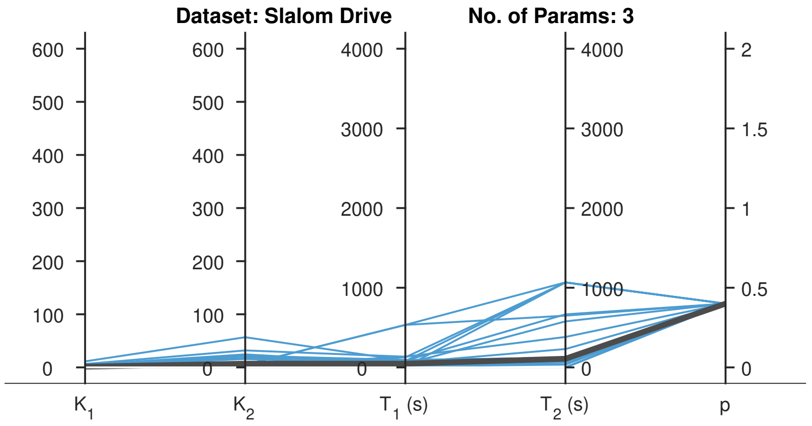

Figure 10a. Slalom drive dataset

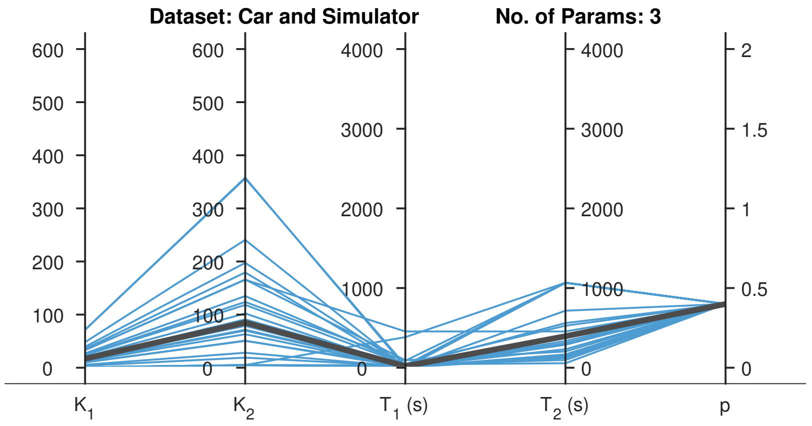

Figure 10b. Car and Simulator dataset

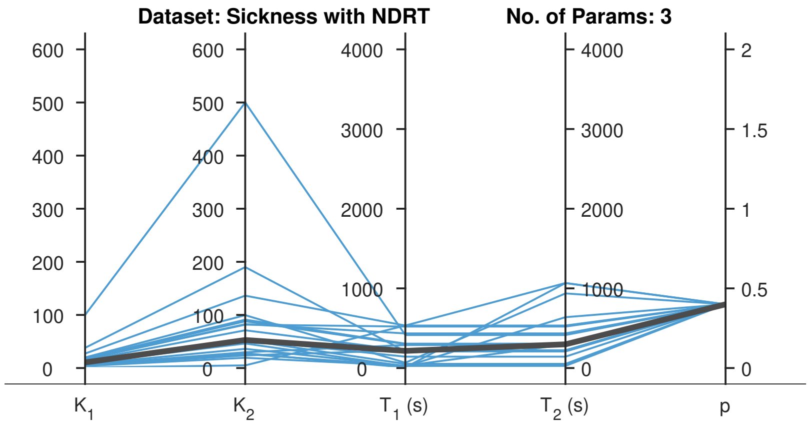

Figure 10c. NDRT Drive dataset

Figure 10. Parameter distribution (estimated gain (K_1) and time constant (T_1)) for the AM3 model (blue) with median values (black) for the three datasets

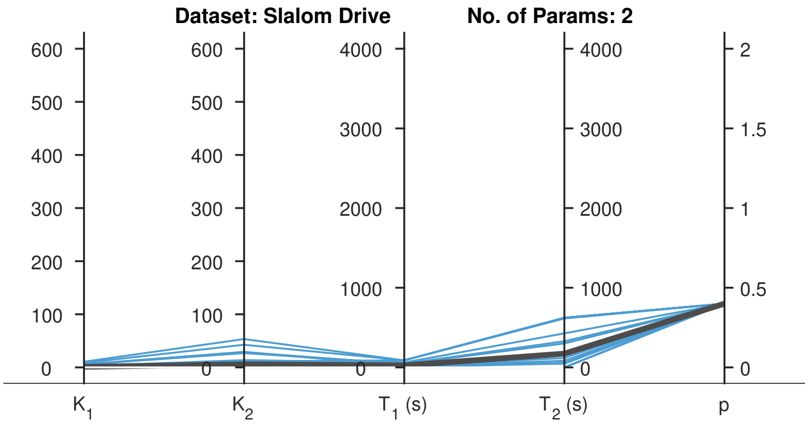

Figure 11a. Slalom drive dataset

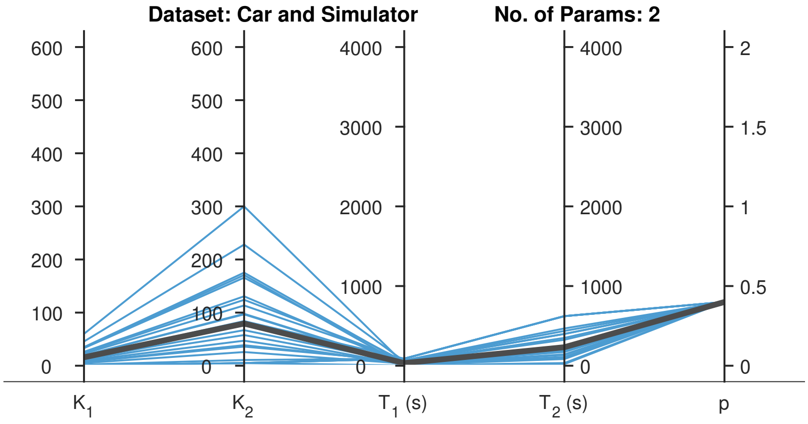

Figure 11b. Car and Simulator dataset

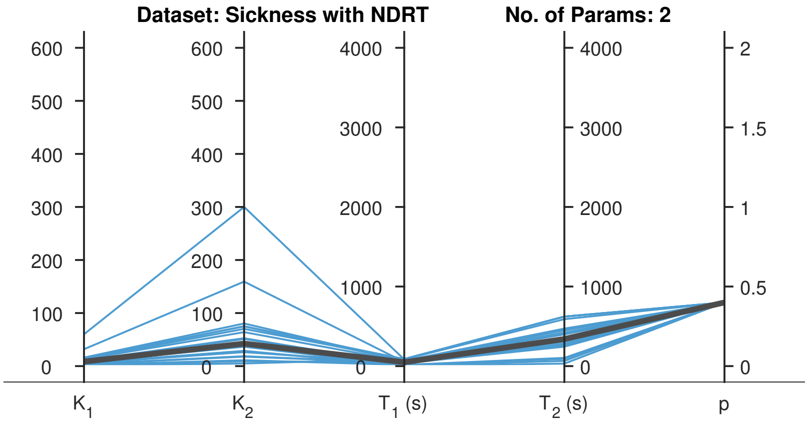

Figure 11c. NDRT Drive dataset

Figure 11. Parameter distribution (estimated gain (K_1) and time constant (T_1)) for the AM2 model (blue) with median values (black) for the three datasets

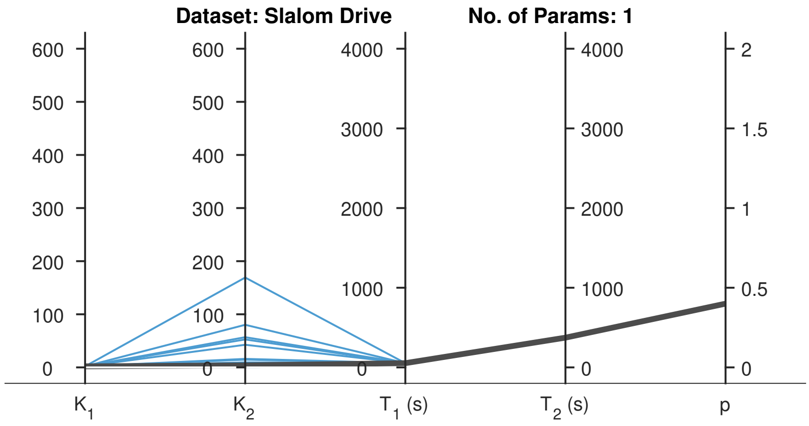

Figure 12a. Slalom drive dataset

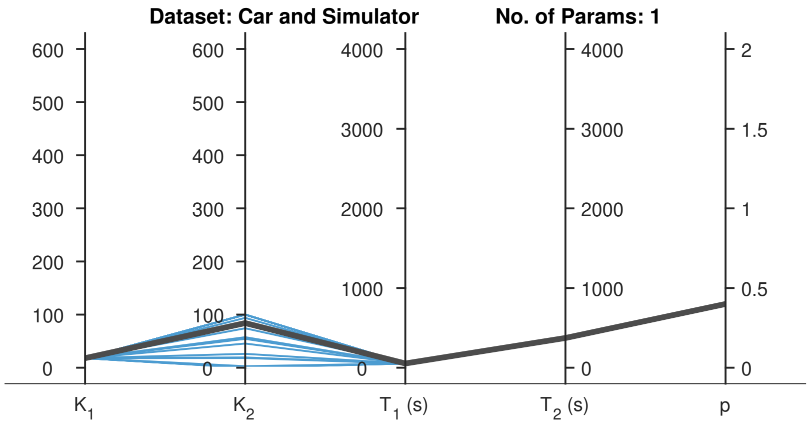

Figure 12b. Car and Simulator dataset

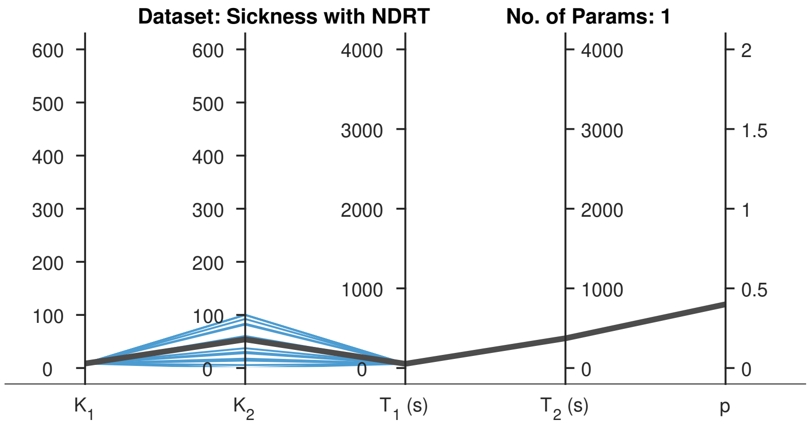

Figure 12c. NDRT Drive dataset

Figure 12. Parameter distribution (estimated gain (K_1) and time constant (T_1)) for the AM1b model (blue) with median values (black) for the three datasets

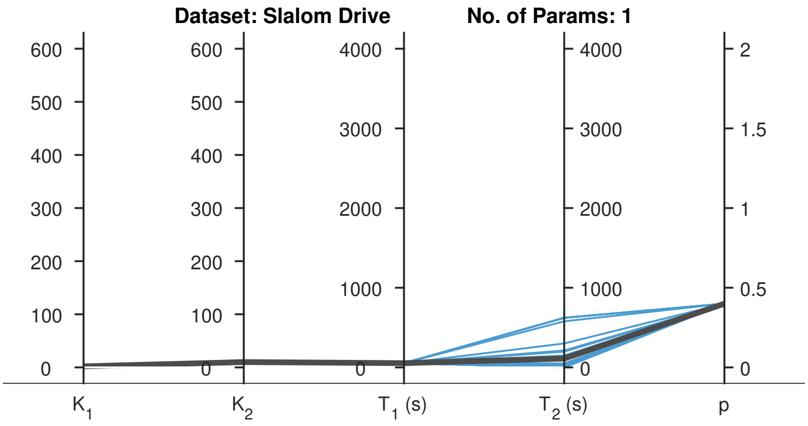

Figure 13a. Slalom drive dataset

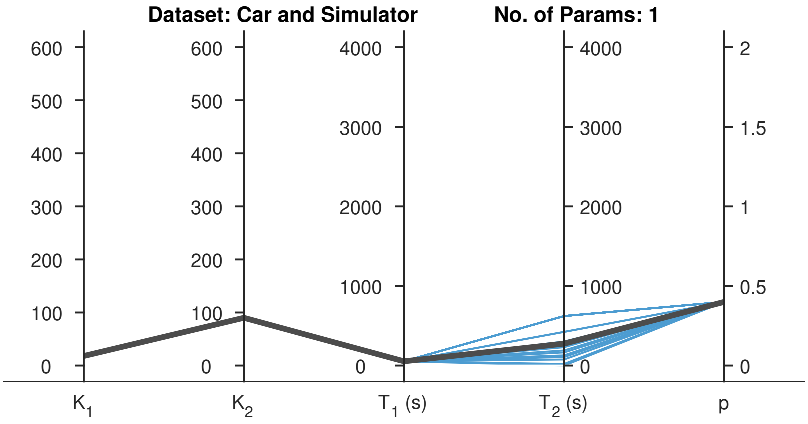

Figure 13b. Car and Simulator dataset

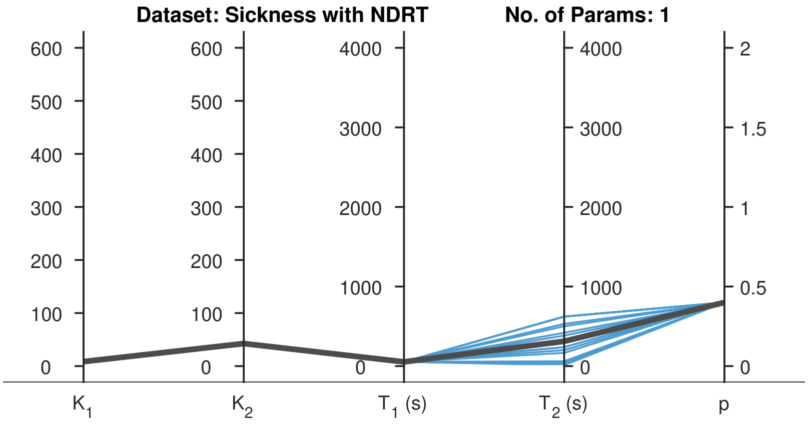

Figure 13c. NDRT Drive dataset

Figure 13. Parameter distribution (estimated gain (K_1) and time constant (T_1)) for the AM1a model (blue) with median values (black) for the three datasets

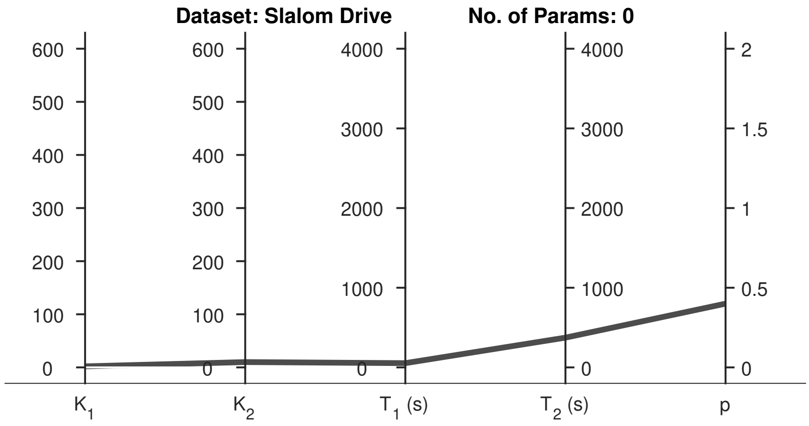

Figure 14a. Slalom drive dataset

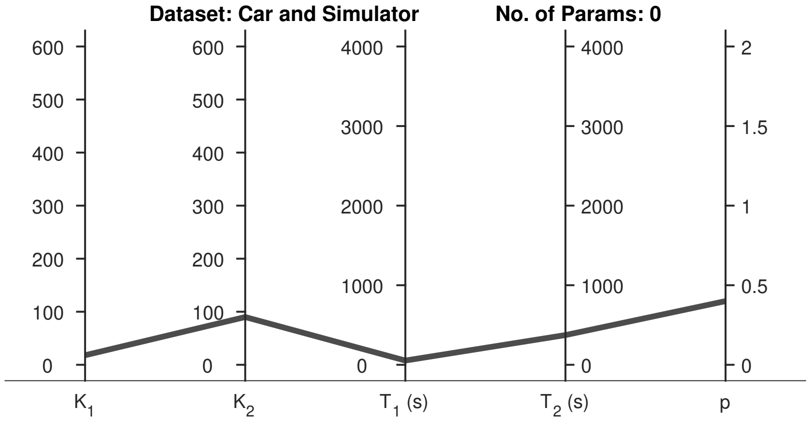

Figure 14b. Car and Simulator dataset

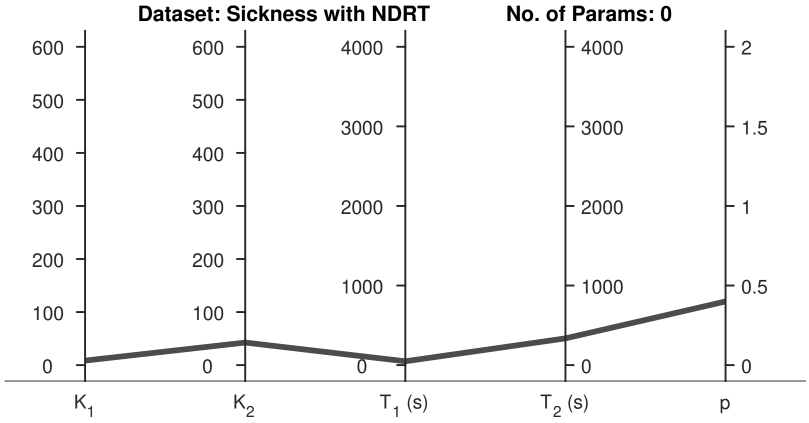

Figure 14c. NDRT Drive dataset

Figure 14. Parameter distribution (estimated gain (K_1) and time constant (T_1)) for the AM0 model (blue) with median values (black) for the three datasets

6 MISERY SCALE (MISC)

The MIsery SCale (MISC) for motion sickness is an established subjective measure from 0 to 10 used to quantify the severity of discomfort experienced during motion sickness based on symptoms such as nausea, dizziness and general feeling of unwell (Bos et al., 2005). It has now been rebranded as the Motion Illness Symptoms Classification (MISC) scale (Reuten et al., 2021).

Table S1. MIsery SCale (MISC) as described in Bos et al. (2005)

Symptoms		Score
No Probliems		0
Slight discomfort but no specific symptoms		1
Dizziness, warm, headache, stomach awareness, sweating, etc.	vage	2
	some	3
	medium	4
	severe	5
Nausea	some	6
	medium	7
	severe	8
	retching	9
Vomiting		10

REFERENCES

Jelte Bos, Ouren X. Kuiper, and Eike A. Schmidt. Motion Predictability and Sickness. In *Driving Simulation Conference Proceedings*, pages 179–182, Antibes, France, 9 2020. Driving Simulation Association.

Jelte E Bos and Williem Bles. Modelling motion sickness and subjective vertical mismatch detailed for vertical motions. *Brain Research Bulletin*, 47(5):537–542, 1998. doi: 10.1016/s0361-9230(98)00088-4.

Takahiro Wada, Junichiro Kawano, Yuki Okafuji, Atsushi Takamatsu, and Mitsuhiro Makita. A Computational Model of Motion Sickness Considering Visual and Vestibular Information. In *2020 IEEE International Conference on Systems, Man, and Cybernetics (SMC)*, pages 1758–1763. IEEE, 10 2020. ISBN 978-1-7281-8526-2. doi: 10.1109/SMC42975.2020.9283350.

Hailong Liu, Shota Inoue, and Takahiro Wada. Motion Sickness Modeling with Visual Vertical Estimation and Its Application to Autonomous Personal Mobility Vehicles. In *2022 IEEE Intelligent Vehicles Symposium (IV)*, pages 1415–1422, 2 2022. ISBN VO -. doi: 10.1109/IV51971.2022.9827161.

Tugrul Irmak, Daan Pool, Ksander de Winkel, and Riender Happee. Validating models of sensory conflict and perception for motion sickness prediction. *Biological Cybernetics*, 3 2023. doi: 10.1007/s00422-023-00959-8.

Varun Kotian, Tugrul Irmak, Daan Pool, and Riender Happee. The role of vision in sensory integration models for predicting motion perception and sickness. *Experimental Brain Research*, 242(3):685–725, 2024. ISSN 1432-1106. doi: 10.1007/s00221-023-06747-x.

Charles M Oman. Motion sickness: a synthesis and evaluation of the sensory conflict theory. *Canadian Journal of Physiology and Pharmacology*, 68(2):294–303, 1990. doi: 10.1139/y90-044.

Tugrul Irmak, Varun Kotian, Riender Happee, Ksander N. de Winkel, and Daan M. Pool. Amplitude and Temporal Dynamics of Motion Sickness. *Frontiers in systems neuroscience*, 16, 5 2022. ISSN 1662-5137. doi: 10.3389/FNSYS.2022.866503.

Jelte Bos, Scott N Mackinnon, and Anthony Patterson. Motion Sickness Symptoms in a Ship Motion Simulator: Effects of Inside, Outside, and No View. *Aviation, Space, and Environmental Medicine*, 76 (12):1111–1118, 12 2005.

Tugrul Irmak, Daan M Pool, and Riender Happee. Objective and subjective responses to motion sickness: the group and the individual. *Experimental Brain Research*, 2020. doi: 10.1007/s00221-020-05986-6.

Tugrul Irmak, Ksander N De Winkel, Daan M Pool, Heinrich H Bülthoff, and Riender Happee. Individual motion perception parameters and motion sickness frequency sensitivity in fore-aft motion. *Experimental Brain Research*, 2021. doi: 10.1007/s00221-021-06093-w.

Varun Kotian, Daan M Pool, and Riender Happee. Modelling individual motion sickness accumulation in vehicles and driving simulators. In *Proceedings of the Driving Simulation Conference*, Antibes, France, 2023.

Tessa M.W. Talsma, Omar Hassanain, Riender Happee, and Ksander N. de Winkel. Validation of a moving base driving simulator for motion sickness research. *Applied Ergonomics*, 106:103897, 1 2023. ISSN 0003-6870. doi: 10.1016/J.APERGO.2022.103897.

Myriam Metzulat, Barbara Metz, Andreas Landau, Alexandra Neukum, and Wilfried Kunde. Does the visual input matter? Influence of non-driving related tasks on car sickness in an open road setting. *Transportation Research Part F: Traffic Psychology and Behaviour*, 104:234–248, 7 2024. ISSN 13698478. doi: 10.1016/j.trf.2024.06.002.

Huseyin Harmankaya, Adrian Brietzke, Rebecca Pham Xuan, Barys Shyrokau, Riender Happee, and Georgios Papaioannou. Efficient Motion Sickness Assessment : Recreation of On-Road Driving on a Compact Test Track. 2024.

Marc Hogerbrug, Joost Venrooij, Daan M. Pool, and Max Mulder. Simulator Sickness Ratings Reduce with Simulator Motion when Driven Through Urban Environments. In *Driving Simulation Conference Proceedings*, pages 175–178, Antibes, France, 9 2020. Driving Simulation Association.

Gerd Baumann, Matthias Jurisch, Christian Holzapfel, Claudia Buck, and Hans-Christian Reuss. Driving simulator studies for kinetosis-reducing control of active chassis systems in autonomous vehicles. In *Driving Simulation Conference Proceedings*, pages 51–58, Munich, Germany, 9 2021. Driving Simulation Association.

Vishrut Jain, Sandeep Suresh Kumar, Georgios Papaioannou, Riender Happee, and Barys Shyrokau. Optimal Trajectory Planning for Mitigated Motion Sickness: Simulator Study Assessment. *IEEE Transactions on Intelligent Transportation Systems*, 2023. ISSN 15580016. doi: 10.1109/TITS.2023.3281724.

Ksander N De Winkel, · Tuğrul Irmak, Varun Kotian, · Daan, M Pool, and · Riender Happee. Relating individual motion sickness levels to subjective discomfort ratings. *Experimental Brain Research 2022*, 1: 1–10, 2 2022. ISSN 1432-1106. doi: 10.1007/S00221-022-06334-6.

Anna J. C. Reuten, Jelte Bos, and Jeroen B. J. Smeets. The metrics for measuring motion sickness. In *Driving Simulation Conference Proceedings*, pages 183–186, Antibes, France, 9 2020. Driving Simulation Association.

Michael McCauley, Jackson Royal, C Wylie, James O'Hanlon, and Robert Mackie. Motion Sickness Incidence: Exploratory Studies of Habituation, Pitch and Roll, and the Refinement of a Mathematical Model. Technical report, Canyon Research Group Inc Goleta Ca Human Factors Research Div., 1976.

John Golding and H. M. Markey. Effect of frequency of horizontal linear oscillation on motion sickness and somatogravic illusion. *Aviation Space & Environmental Medicine*, 67(2):121–126, 1996.

Michael J Griffin and Kim L Mills. Effect of frequency and direction of horizontal oscillation on motion sickness. *Aviation, space, and environmental medicine*, 73(6):537–543, 6 2002. ISSN 0095-6562 (Print).

Henrietta V C Howarth and Michael J Griffin. Effect of roll oscillation frequency on motion sickness. *Aviation, space, and environmental medicine*, 74(4):326–331, 4 2003. ISSN 0095-6562 (Print).

Diane Cleij, Joost Venrooij, Paolo Pretto, Daan M. Pool, Max Mulder, and Heinrich H. Bülthoff. Continuous subjective rating of perceived motion incongruence during driving simulation. *IEEE Transactions on Human-Machine Systems*, 48(1):17–29, 2 2018. ISSN 21682291. doi: 10.1109/THMS.2017.2717884.

Maurice Kolff, Markus Schwienbacher, Joost Venrooij, Daan M Pool, and Max Mulder. Motion Cueing Quality Comparison of Driving Simulators using Oracle Motion Cueing. In *Driving Simulation Conference Proceedings*, Strasbourg, 9 2022.

Hirotogu Akaike. Information Theory and an Extension of the Maximum Likelihood Principle. *Biogeochemistry*, 1998:199–213, 1998. ISSN 1573515X. doi: 10.1007/978-1-4612-1694-0{_}15.

Gideon Schwarz. Estimating the Dimension of a Model. *https://doi.org/10.1214/aos/1176344136*, 6(2): 461–464, 3 1978. ISSN 0090-5364. doi: 10.1214/AOS/1176344136.

Norimasa Kamiji, Yoshinori Kurata, Takahiro Wada, and Shun'ichi Doi. Modeling and validation of carsickness mechanism. In *Proceedings of the SICE Annual Conference*, pages 1138–1143. IEEE, 9 2007. ISBN 4907764286. doi: 10.1109/SICE.2007.4421156.

Frank M. Drop, Daan M. Pool, Marinus M. Van Paassen, Max Mulder, and Heinrich H. Bülthoff. Objective Model Selection for Identifying the Human Feedforward Response in Manual Control. *IEEE transactions on cybernetics*, 48(1):2–15, 1 2018. ISSN 2168-2275. doi: 10.1109/TCYB.2016.2602322.

Michael C Newman. *A Multisensory Observer Model for Human Spatial Orientation Perception*. PhD thesis, Massachusetts Institute of Technology, 6 2009.

Torin K. Clark, Michael C. Newman, Faisal Karmali, Charles M. Oman, and Daniel M. Merfeld. Mathematical models for dynamic, multisensory spatial orientation perception. *Progress in Brain Research*, 248:65–90, 2019. doi: 10.1016/BS.PBR.2019.04.014.

International Organization For Standardization. ISO 2631-1:1997 - Mechanical vibration and shock - Evaluation of human exposure to whole-body vibration - Part 1: General requirements, 1997.

Daofei Li and Jiankan Hu. Mitigating Motion Sickness in Automated Vehicles with Frequency-Shaping Approach to Motion Planning. *IEEE Robotics and Automation Letters*, 6(4):7714–7720, 10 2021. ISSN 23773766. doi: 10.1109/LRA.2021.3101050.

Raj Desai, Marko Cvetković, Georgios Papaioannou, and Riender Happee. Evaluation of Motion Comfort using Advanced Active Human Body Models and Efficient Simplified Models. *IEEE Conference on Intelligent Transportation Systems, Proceedings, ITSC*, pages 5351–5356, 2023. ISSN 21530017. doi: 10.1109/ITSC57777.2023.10422474.

Riender Happee, Varun Kotian, and Ksander de Winkel. Neck stabilization through sensory integration of vestibular and visual motion cues. *Frontiers in Neurology*, 14:1266345, 2023. ISSN 1664-2295. doi: 10.3389/FNEUR.2023.1266345.

Anna J. C. Reuten, Suzanne A. E. Nooij, Jelte E. Bos and Jeroen B. J. Smeets How feelings of unpleasantness develop during the progression of motion sickness symptoms. *Experimental Brain Research*, 239(12) pages 3615–3624 doi: 10.1007/s00221-021-06226-1.
